# Spliceosome SNRNP200 Promotes Viral RNA Sensing and IRF3 Activation of Antiviral Response

**DOI:** 10.1371/journal.ppat.1005772

**Published:** 2016-07-25

**Authors:** Nicolas Tremblay, Martin Baril, Laurent Chatel-Chaix, Salwa Es-Saad, Alex Young Park, Robert K. Koenekoop, Daniel Lamarre

**Affiliations:** 1 Centre de Recherche du CHUM (CRCHUM), Montréal, Québec, Canada; 2 Faculté de Médecine, Université de Montréal, Montréal, Canada; 3 Departments of Pediatric Surgery, Human Genetics and Ophthalmology, McGill University, Montréal, Canada; Mount Sinai School of Medicine, UNITED STATES

## Abstract

Spliceosomal SNRNP200 is a Ski2-like RNA helicase that is associated with retinitis pigmentosa 33 (RP33). Here we found that SNRNP200 promotes viral RNA sensing and IRF3 activation through the ability of its amino-terminal Sec63 domain (Sec63-1) to bind RNA and to interact with TBK1. We show that SNRNP200 relocalizes into TBK1-containing cytoplasmic structures upon infection, in contrast to the RP33-associated S1087L mutant, which is also unable to rescue antiviral response of SNRNP200 knockdown cells. This functional rescue correlates with the Sec63-1-mediated binding of viral RNA. The hindered IFN-β production of knockdown cells was further confirmed in peripheral blood cells of RP33 patients bearing missense mutation in SNRNP200 upon infection with Sendai virus (SeV). This work identifies a novel immunoregulatory role of the spliceosomal SNRNP200 helicase as an RNA sensor and TBK1 adaptor for the activation of IRF3-mediated antiviral innate response.

## Author Summary

The innate immune system is the first line of defense against pathogens and relies on the recognition of molecular structures specific to pathogens by sensor receptors. These receptors activate a signaling cascade and induce a protective cellular innate immune response. In this study, we provide evidence for a role of the spliceosomal SNRNP200 RNA helicase in promoting antiviral response that is clearly distinguishable of the one in pre-mRNA splicing. The depletion of SNRNP200 in human cells resulted in a reduced interferon-β (IFNB1) production and increased susceptibility to viral infection. We showed that SNRNP200 positively regulates activation of the key transcription factor IRF3 via interaction with TANK kinase 1 (TBK1). Upon infection, SNRNP200 binds viral RNA and relocalizes into TBK1-containing cytoplasmic structures to promote IRF3 activation and IFNB1 production. Of clinical relevance, we observed a significantly hindered antiviral response of PBMCs from patients carrying a dominant SNRNP200 mutation associated with retina pigmentosa type 33 (RP33), an inherited degenerative eye disease. We showed that the RP33-associated S1087L SNRNP200 mutant has lost the ability to bind RNA and that its expression fails to rescue antiviral response in SNRNP200 silenced cells. Our study provides new insights into a role within the antiviral response for spliceosome SNRNP200 helicase as an RNA sensor and TBK1 adaptor in IRF3 signaling.

## Introduction

The innate immune system is the first line of defense against pathogens, and it relies on the recognition of pathogen-associated molecular patterns (PAMPs) by specific pattern recognition receptors (PRRs). Upon viral infection, intracellular foreign nucleic acids are detected by specific DExD-box RNA helicases of the RIG-I-like receptor (RLRs) family: RIG-I (also known as DDX58), MDA5 (also known as IFIH1), and LGP2 (also known as DHX58) [1]. In response to sensing viral RNA, these RLRs associate with the MAVS adaptor (also called IPS-1, Cardif, and VISA) [2–5] to induce its multimerization [6,7] and to activate multiple kinases, including IKK, TBK1, and IKBKE. Upon signal transduction, the activation of transcription factors such as AP-1 (also known as ATF-2/c-jun), NF-κB, IRF3, and IRF7 induces the expression of pro-inflammatory and antiviral cytokines and chemokines. Type I Interferons (IFNs) then trigger the activation of STAT1, STAT2, and IRF9, forming a transcription factor complex known as IFN-stimulated gene factor 3 (ISGF3) to ultimately induce a large number of IFN-stimulated genes (ISGs). In a recent genome-wide RNAi screening that assessed virus-induced IFNB1 transcription [8], spliceosomal factors, including the SNRNP200 RNA helicase, that positively modulate the RLR-mediated antiviral pathway were identified. Few studies have described a contribution of spliceosomal factors in pathogen-mediated immune responses, though studies have examined the effects of alternative mRNA splicing of innate immunity genes, such as DDX58, MyD88, and IRF3 [9–11]. Interestingly, many DExD/H-box RNA helicases were recently identified as viral nucleic acids sensor and/or mediator components of antiviral innate immunity [12,13]. DHX15 and DHX9 helicases were shown to interact with MAVS, following dsRNA recognition, and to activate NF-κB, IRF3, and MAPK pathways in myeloid dendritic cells (mDC) [12,14]. An RNA helicase complex composed of DDX1, DDX21, and DHX36 was reported to induce type I IFN through TRIF-dependent signaling in mDC [15]. Two other helicases, DDX60 and DDX3, were shown to bind DDX58/MDA5 and to enhance its recognition of dsRNA while also enhancing downstream type I IFN production [16,17]. DDX3 acts as an adaptor protein of TBK1 and IKBKE, thereby synergistically enhancing IFNB1 promoter induction [18,19]. Finally, DDX41 helicase is a DNA sensor that activates type I IFN via a STING-TBK1 complex [20]. In the present study, it was found that silencing SNRNP200, a core spliceosome RNA helicase and unique member of the Ski2-like subfamily, leads to a strong decrease in the antiviral innate response by positively regulating IRF3 signaling upon Sendai virus (SeV) infection. In SNRNP200 knockdown (KD) cells, unlike the expression of wild-type (WT) protein, expression of the S1087L variant associated with retinitis pigmentosa 33 (RP33) is unable to rescue IFNB1 transcription. The functional rescue phenotype correlates with the ability of the amino-terminal Sec63 domain (Sec63-1) of SNRNP200 to bind surrogate polyinosinic-polycytidylic acid (poly I:C) and viral RNA. For instance, upon infection by SeV, viral RNA allows SNRNP200 to relocalize into TBK1-containing cytosolic structures. A physical interaction between SNRNP200 and TBK1 was also observed, and this interaction was mapped to the Sec63-1 domain. Finally, a significantly hindered antiviral response was demonstrated in human monocyte-derived macrophages (MDM) silenced for SNRNP200 and in peripheral blood cells (PMBCs) from RP33 patients with pathogenic missense S1087L mutation in SNRNP200. Ultimately, this study revealed a novel immunoregulatory role of spliceosome SNRNP200 helicase in viral RNA sensing and in promoting IRF3-dependent antiviral innate immune responses.

## Results

### Identification of spliceosome SNRNP200 required for SeV-induced IFNB1 transcription

**Fig 1:**
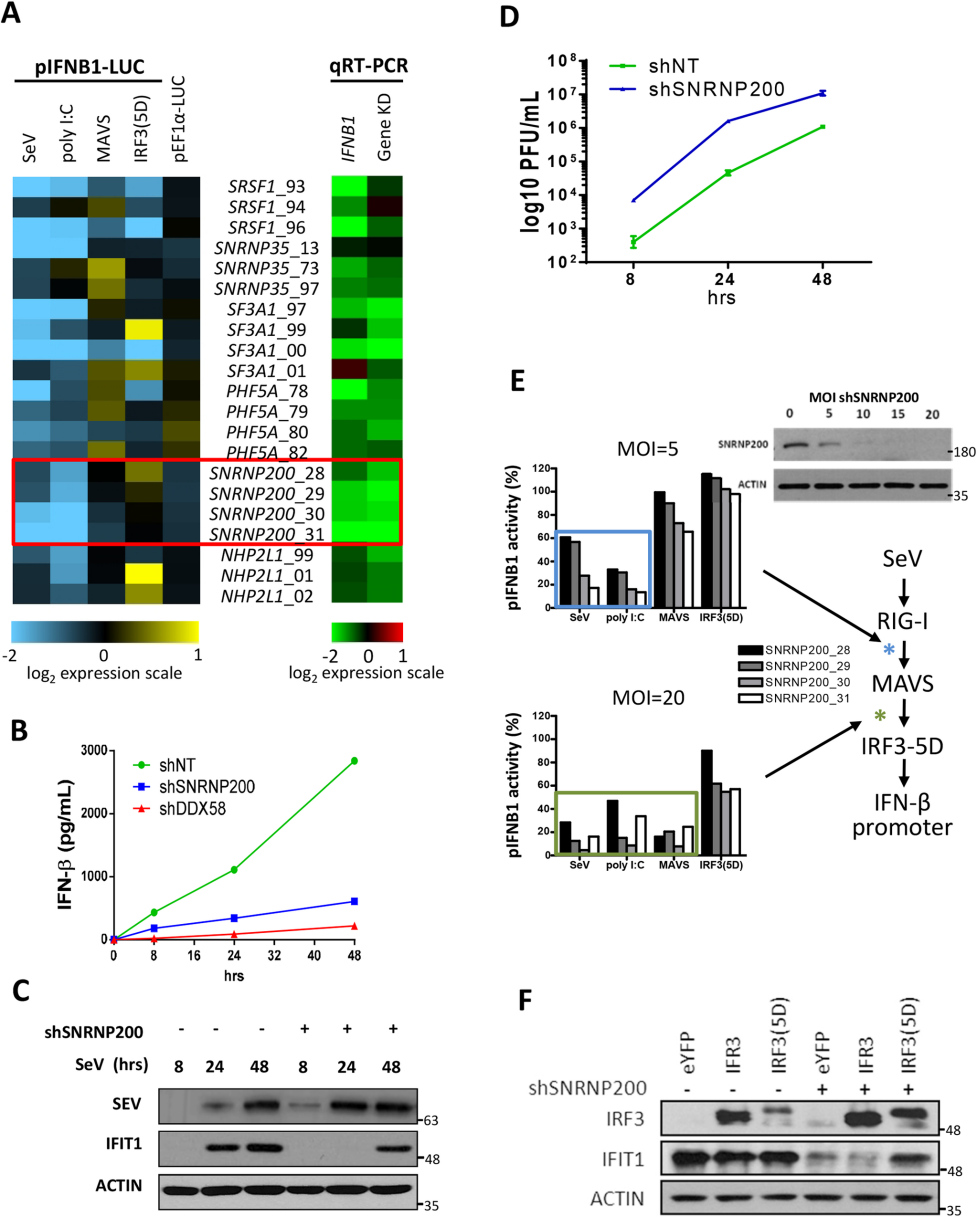
SNRNP200 spliceosome protein is required for virus-induced IFNB1 production to control viral replication. (A) HEK 293T cells stably expressing an IFNB1promoter-driven luciferase gene (HEK 293T pIFNB1-Luc) are transduced with different lentiviral-expressing shRNA targeting SNRNP200, SFRS1, SNRNP35, SF3A1, PHF5A and NHP2L1 genes. Left panel—Heat map (log2 scale) indicating the modulation of IFNB1 promoter activity following silencing of spliceosome genes and infection with SeV or transfection of poly I:C, MAVS or IRF3(5D) expression plasmids for 16 hours. Right panel—qRT-PCR validation data of the endogenous IFNB1 mRNA levels and target gene KD efficiency of cells transduced with shRNA. (B) HEK 293T are transduced with lentiviral-expressing shRNA control (shNT) or targeting SNRNP200 (shSNRNP200) or DDX58 (shDDX58) for three days and infected cells with SeV for 8, 24 or 48 hours. Supernatants are harvested and IFN-β secretion levels are measured by ELISA. (C) Immunoblot analysis of HEK 293T cells infected with SeV for 8, 24 or 48 hours following treatment with shNT or shSNRNP200 for three days. SeV, IFIT1 and actin proteins are resolved by immunobloting at the indicated time. (D) Infectivity titers of SeV particles produced as indicated in (C) are determined by harvesting supernatants at the indicated time and infecting VERO cells in virus plaque assays. (E) HEK 293T pIFNB1-Luc cells are transduced with four different shSNRNP200 at a multiplicity of infection (MOI) of 5 and 20 for three days. Relative IFNB1 promoter activity are reported as percentage of the control shRNA NT after infection with SeV or transfection of poly I:C, MAVS or IRF3(5D) expression plasmids for 16 hours (left). Simplified schematic of RLR signaling pathway leading to IFN-β promoter induction (right). Deduced points of action of SNRNP200 are marked with asterisks (blue and green for MOI = 5 and 20, respectively). Knockdown efficiencies at the various MOI are determined by immunobloting analysis of SNRNP200 protein levels. (F) Immunoblot analysis of HEK 293T cells transduced with shNT or shSNRNP200 for three days and subjected to SeV infection for 16 hours. Plasmids encoding eYFP, IRF3 and IRF3(5D) are transfected for 48 hours. Following cell harvesting, IRF3 and IFIT1 protein levels are resolved by immunobloting analysis of cell lysates.

A genome-wide gene silencing screen that assessed the transcriptional activity of the IFNB1 promoter following SeV infection was previously performed to identify novel regulators of innate immunity [8]. Six genes that encode spliceosome components that reduced IFNB1 transcription upon gene silencing were identified (Fig 1A). Among these genes, one encodes an RNA helicase (SNRNP200) and two (SF3A1 and SRSF1) were shown to regulate innate immune responses by the alternative splicing of either Myd88 or IRF3 [10,11]. To further explore RNA helicases that play central roles in splicing and that often function in proofreading events in pre-mRNA splicing [21], an RNAi mini-screen using five independent lentiviruses that express short hairpin RNA (shRNA) targeting most spliceosomal RNA helicases was performed (S1A Fig). SNRNP200 was the only RNA helicase assigned to the Ski2-like helicase subfamily that showed a significant reduction in IFNB1 promoter-driven reporter activity. The KD of SNRNP200 was validated through the marked decrease in the mRNA and the protein levels while its specificity was validated by the absence of any off-target effects on other spliceosome gene hits (S1B and S1C Fig). The depletion of SNRNP200 reduced IFN-β production at 8 hours post-infection reaching levels comparable to those obtained in DDX58 KD cells at 48 hours post-infection (Fig 1B). Interestingly, while the depletion of SNRNP200 completely inhibited IFIT1 (also known as ISG56) induction (S1C Fig), its overexpression could not increase IFIT1 levels in neither the non-infected nor the SeV-infected cells. To further investigate SNRNP200’s contribution to the antiviral response, the viral susceptibility of SNRNP200 KD cells was monitored in a time-course experiment by following IFIT1 induction along with the production of infectious particles and the viral protein levels (Fig 1C and 1D). In control HEK 293T cells transduced with a non-target sequence shRNA (shNT)-expressing lentivirus, SeV protein was only detectable at 24 hours post-infection which coincided with IFIT1 induction (Fig 1C). In contrast, in SNRNP200 KD cells, SeV protein was readily detectable at 8 hours post-infection becoming more significant at 24 hours post-infection. However, IFIT1 induction was only detected at 48 hours post-infection. Of notable importance, SNRNP200 KD cells were observed to yield up to a 2-log increase in viral titers when compared to the control (Fig 1D). Correlating with a reduced early IFNB1 induction, it was confirmed that silencing SNRNP200 also increased the replicative potential of influenza A virus (FLUA) and hepatitis C virus (HCV) in HEK 293T and Huh7 cells, respectively (S2 Fig). Based on the epistasis analysis, the transcriptional activity of the IFNB1 promoter is slightly affected by ectopic expression of constitutively active IRF3(5D) [22], while it was completely blocked in SeV infected or MAVS overexpressing cells transduced at a high multiplicity of infection (MOI of 20) with lentiviral-expressing shRNA (Fig 1E). Similar results were obtained in A549 cells (S3A and S3B Fig). Interestingly, upon SeV infection and in contrast to IRF3 overexpression, the ectopic expression of IRF3(5D) could rescue the induction of IFIT1 (Fig 1F), suggesting a role for SNRNP200 in IRF3 activation. The effect of SNRNP200 KD in NF-κB-dependent transcription was then investigated using a reporter assay (p2xNF-κB_fLUC) in HEK 293T cells. It was found that SNRNP200 KD cells display no attenuation of poly (I:C)-, MAVS-, TBK1-, or p65-mediated activation of the NF-κB promoter (S4A Fig). In contrast, in these cells, there was a significant inhibition of SeV-, poly (I:C)-, TBK1-, and IFN-α-mediated activation of the ISG56 promoter (S4B Fig). It was confirmed that SNRNP200 silencing does not affect NF-κB-dependent transcription in SeV-infected A549 cells through the quantification of TNF, NFKBIA, and TNFAIP3 mRNA levels using qRT-PCR (S3C Fig). Interestingly, neither the TRIF nor the cGAS/STING pathways are affected in SNRNP200 KD cells whereas the RLR pathway, which converge to the TBK1-mediated phosphorylation of their respective adaptors (TRIF, STING, and MAVS) to recruit IRF3 and to license IRF3 for phosphorylation [23]. These data suggest that, upon RNA virus infection, SNRNP200 may function between MAVS signal transduction and TBK1-mediated IRF3 licensing. These observations led to exploring a specific regulatory role of SNRNP200, a core component of U4/U6-U5 small nuclear RNA (snRNA) [24], in the downstream activation of IRF3, of production of IFNB1 and ultimately of an optimal antiviral response.

### SNRNP200 specifically regulates IRF3 signaling upon RNA virus infection

**Fig 2:**
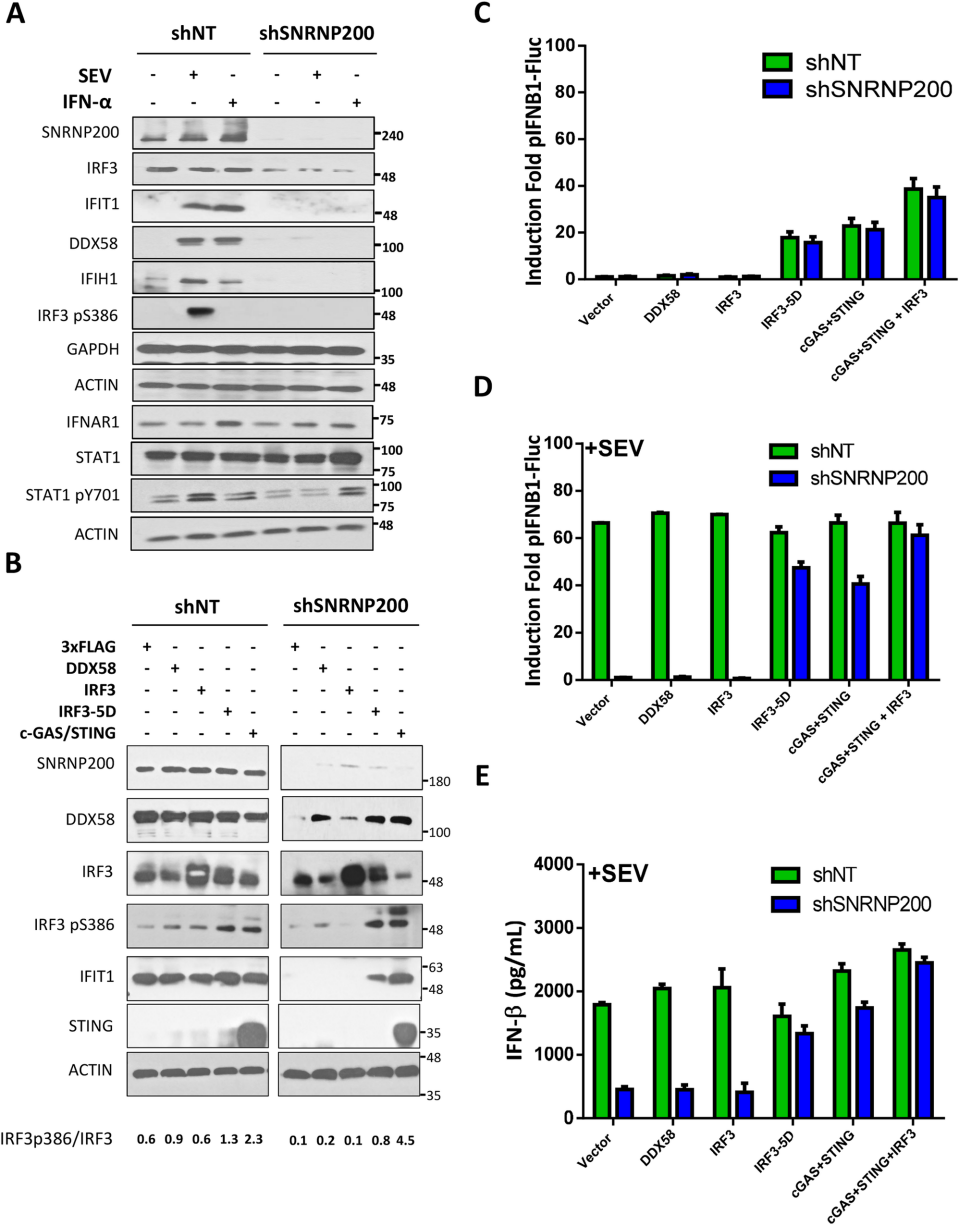
SNRNP200 KD restricts SeV- and type I IFN-mediated induction of antiviral response. (A) HEK 293T cells are transduced with shNT or shSNRNP200 and infected with SeV or stimulated with IFN-α for 24 hours. Selected genes are resolved by immunobloting and compared to shNT control cells. (B) HEK 293T are transduced with shNT or shSNRNP200 for 3 days and transfected with DDX58, IRF3, IRF3(5D) and cGAS-STING expression plasmids for the last 48 hours, and subsequently infected with SeV for 24 hours. Selected genes are resolved by immunobloting and compared with cells transduced with shNT. (C) HEK 293T pIFNB1-Luc cells are transduced with shNT or shSNRNP200 for 3 days and transfected with DDX58, IRF3, IRF3(5D) and cGAS-STING expression plasmids for the last 48 hours. Luciferase levels are resolved and compared to shNT control cells. (D) HEK 293T pIFNB1-Luc cells are transduced with shNT or shSNRNP200 for 3 days and transfected with DDX58, IRF3, IRF3(5D) and cGAS-STING expression plasmids for the last 48 hours, and subsequently infected with SeV for 24 hrs. Luciferase levels are resolved and compared to shNT cells. (E) HEK 293T cells are treated as indicated in D. At 24 hrs post-infection, supernatants are harvested and IFN-β secretion levels are measured by ELISA.

To understand the manner in which SNRNP200, upon viral infection, contributes to IRF3-mediated IFNB1 production, the effect of SNRNP200 silencing on the expression of established members of the RLR pathway was evaluated using a western blot analysis (S5A Fig). First, a decreased protein expression of IRF3 in SNRNP200 KD cells was observed. This correlated with the blockage of the SeV-mediated induction of IFIT1, DDX58, and IFIH1 proteins. The decreased IRF3 protein levels were further confirmed at the mRNA level by qRT-PCR, paralleling the reduced mRNA levels of SNRNP200 and its effector genes (IFNB1, IFIT1, DDX58, and IFIH1) (S5B Fig). While the residual IRF3 protein levels of KD cells are sufficient for the activation of the cGAS/STING pathway (S4B Fig), a complete inhibition of IRF3 phosphorylation at serine 386 following SeV infection (Fig 2A, see IRF3-p386) was observed. This suggested a specific contribution of SNRNP200 during IRF3-activation-mediated IFNB1 production. A weak decrease of the basal protein expression levels of DDX58 in SNRNP200 KD cells was also observed (S5A Fig). The mRNA levels, however, were comparable to the control shNT cells (S5B Fig), suggesting that SNRNP200 enhances the RLR-mediated antiviral signaling potential of DDX58 at the basal level. In contrast, the protein expression of MAVS, TBK1, IKBKE, RELA (p65), and TRAF3, which all contribute to the signal propagation for IFNB1 induction, remained unchanged in all conditions (S5A Fig). Similar observations were made for the protein expression of the housekeeping genes ACTIN, TUBULIN, and GAPDH (S5A Fig). To better evaluate the outcome of the reduced basal protein levels of DDX58 and IRF3 on IFNB1 production, ectopic expression of DDX58 and IRF3 in SNRNP200 KD cells was used in an attempt to restore antiviral response. Surprisingly, overexpression of DDX58 and IRF3 neither alone nor in combination could restore SeV-mediated IFIT1 induction (Fig 2B and S6A and S6B Fig). Furthermore, ectopic expression of neither DDX58 nor IRF3 restored IFNB1 promoter reporter activity or IFN-β cytokine production upon SeV infection (Fig 2D and 2E). This was in stark contrast to the almost complete rescue achieved by the ectopic expression of IRF3(5D) (Fig 2D and 2E). The phosphorylation of IRF3 at serine 386 (IRF3-p386) was also noted as a key event for IRF3 activation and, as such, the proportion of IRF3-p386 in relation to total IRF3 was investigated. The quantitative ratios of IRF3-p386 to total IRF3 in context of endogenous or overexpressed DDX58, IRF3, IRF3(5D), and cGAS/STING were determined (Fig 2B). When comparing control shNT-treated with SNRNP200 KD cells in the context of SeV-mediated infection, regardless of whether or not DDX58 or IRF3 was overexpressed, a significant reduction in the IRF3-p386/IRF3 ratios (from 0.6–0.9 to 0.1–0.2) was observed (Fig 2B). This establishes the requirement of SNRNP200 for downstream IRF3 activation independent of its effects on protein expression. Furthermore, ectopic expression of IRF3(5D) in SNRNP200 KD cells yielded IRF3-p386/IRF3 ratios comparable to those of the control shNT-treated cells (0.8 vs 0.6–1.3) correlating with the almost complete restoration of SeV-mediated IFNB1 production. The effect of SNRNP200 KD on the DNA sensing arm of the antiviral response downstream of cGAS/STING was also investigated. It was shown that SNRNP200 is dispensable for cGAS/STING-mediated IFIT1 induction, IFN-β production, and IFNB1 promoter activity (Fig 2B, 2D and 2E), further implying a specific role for SNRNP200 in the RLR-mediated IRF3 signaling pathway upon RNA virus infection. Interestingly, the higher IRF3-p386/IRF3 ratios (4.5) in SNRNP200 KD cGAS/STING expressing cells versus control cells (2.3) largely reflects a significant increase in IRF3 activation and hence in IFIT1 induction (Fig 2B). This suggests that SNRNP200 potentially competes with the STING adaptor during TBK1-mediated IRF3 phosphorylation. Although IRF3 expression slightly increased IFN-β secretion and the IFNB1 promoter activity when activation of the cGAS/STING pathway and SeV infection were combined in SNRNP200 KD cells (Fig 2D and 2E), similar IFNB1 induction was observed in uninfected cells (Fig 2C), demonstrating that IRF3 protein levels in SNRNP200 KD cells have little functional consequence on the cytosolic DNA sensing pathway. Finally, the IRF3 mRNA splice junctions and the presence of exons were investigated to explain the reduced mRNA and protein levels of IRF3. No splicing variants were observed excluding an alternative splicing regulation of IRF3 and supporting a reduction in the efficiency of pre-mRNAs splicing to explain the phenotype of SNRNP200 KD cells (S7 Fig).

Interestingly, the induction of IFIT1, DDX58, and IFIH1 was also inhibited upon treatment of SNRNP200 KD cells with IFN-α (Fig 2A). Furthermore, similar levels of IFNα/β receptor alpha chain (IFNAR1), of STAT1, and of phosphorylation at tyrosine 701 (STAT1pY701) were observed between control and SNRNP200 KD cells following stimulation with IFN-α. This suggests the involvement of SNRNP200 at a later stage of type I IFN signaling downstream of STAT1 phosphorylation. The negative effect of SNRNP200 KD on IFN-α signaling was also demonstrated by the reduced mRNA levels of IFN inducible genes IFIT1, DDX58, and IFIH1 in A549 cells (S3C Fig).

**Fig 3:**
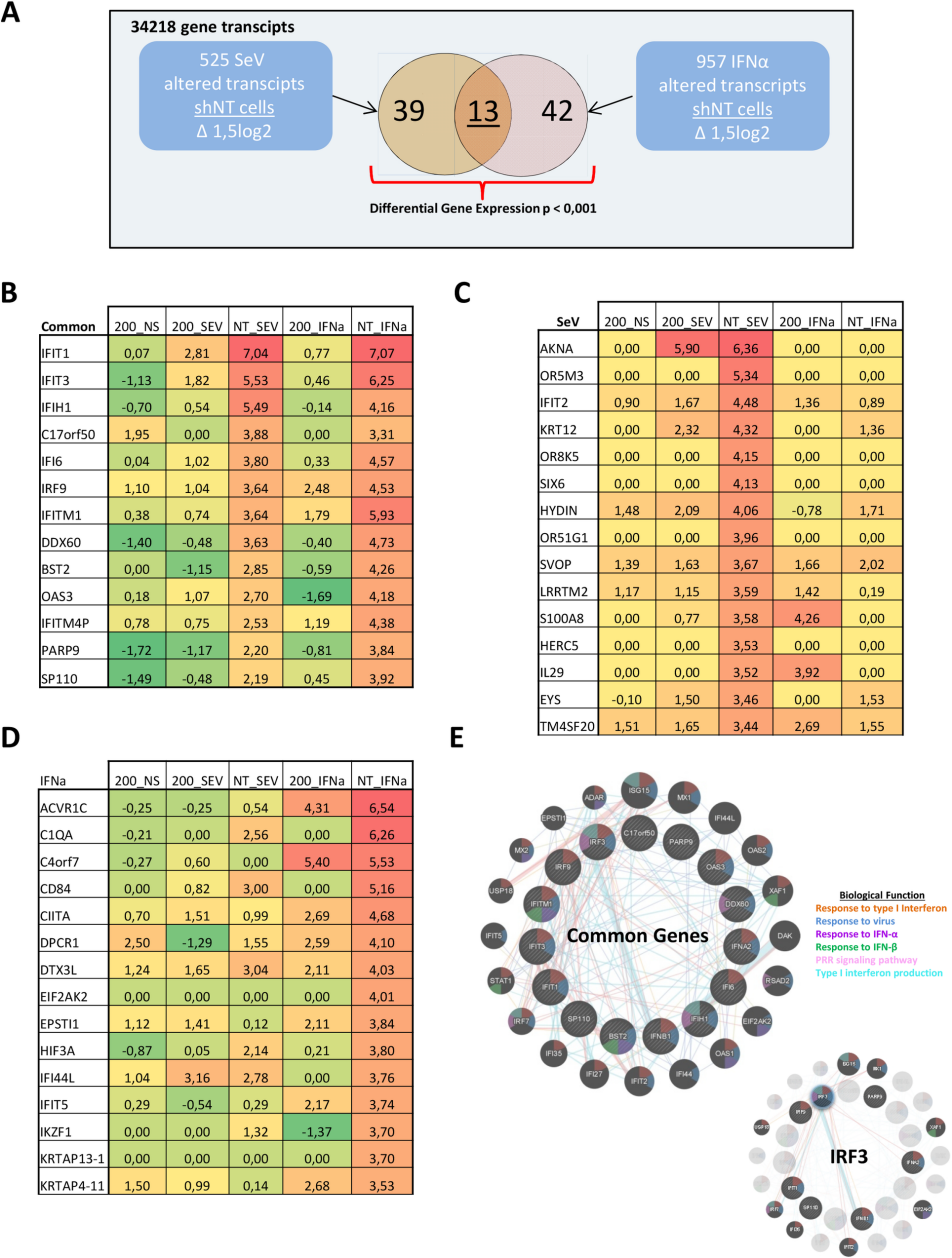
Transcriptional profiles of SNRNP200 KD cells reveal altered expression of genes induced by SeV infection and IFN-α treatment. A) Summary diagram of the transcriptional analysis of shNT control cells to illustrate that out of the 525 SeV altered transcripts and 957 IFN-α altered transcripts, 52 and 55 genes have a differential gene expression (p ≥ 0.001) upon SeV infection or IFN-α stimulation, respectively. (B) Differential gene expression of the 13 transcripts affected by SeV infection and IFN-α stimulation. Expression Fold Change are shown for shSNRNP200 cells (200_NS), shSNRNP200 cells + SeV (200 SEV) and shSNRNP200 cells + IFN-α (200_IFNa) and for shNT control cells (NT_SEV, NT_IFNa). Numerical values are log2 fold change. (C) Differential gene expression of the 39 transcripts affected by SeV infection. Expression Fold Change are shown for shSNRNP200 cells (200_NS), shSNRNP200 cells + SeV (200 SEV) and shSNRNP200 cells + IFN-α (200_IFNa) and for shNT control cells (NT_SEV, NT_IFNa). Numerical values are log2 fold change. Top 15 genes are displayed. Complete list and gene network are available in supporting information (S1 Fig and S1 Table). (D) Differential gene expression of the 42 transcripts affected by IFN-α stimulation. Expression Fold Change are shown for shSNRNP200 cells (200_NS), shSNRNP200 cells + SeV (200 SEV) and shSNRNP200 cells + IFN-α (200_IFNa) and for shNT control cells (NT_SEV, NT_IFNa). Numerical values are log2 fold change. Top 15 genes are displayed. Complete list and gene network are available in supporting information. (E) Interaction network of the 13 common genes shown in (a) and affected by SNRNP200 silencing. The colors inside the dot represent their biological processes (see legend on left). Lines represent physical interactions (protein-protein interactions), pathways (blue), and co-localization (purple) attributes. Shaded nodes represent input data; Black nodes represent most likely first degree interactor. Higher magnification and input genes of this network can be found in supporting documentation. The network on the right corner represents the connections to IRF3.

To comprehensively understand the effect of SNRNP200 KD on type I IFN production (early) and signaling (late), expression profiling studies of non-stimulated (NS), SeV-infected and IFN-α-treated SNRNP200 KD cells versus control shNT HEK 293T cells were performed to assess differential gene expression (Fig 3 and S8 Fig). The effect of SNRNP200 KD on basal gene expression of NS cells was characterized; 2,880 altered transcripts (cutoff of 1,5 log2 fold induction) that are primarily associated with immune system functions and cell cycle regulation were found based on a Reactome pathway enrichment analysis (S8A Fig). A list of transcriptionally altered genes by SeV infection or by IFN-α stimulation in control shNT cells was then established. Using a stringent significance cutoff (p ≤0,001), 52 genes altered by SeV infection and 55 genes altered by IFN-α stimulation were found to be transcriptionally affected by SNRNP200 (Fig 3A). Within these subsets of genes, 13 were in common reflecting the expected overlap of the early (SeV-mediated IFNB1 production) and the late (type I IFN signaling) arms of the antiviral response. Within the subset of commonly affected genes, all showed altered expression upon SNRNP200 silencing with a mean difference of 3,8 log2 fold change when compared to the shNT control cells (Fig 3B). On the other hand, the two subsets of 39 SeV-specific and 42 IFN-α specific genes were hindered by SNRNP200 silencing by a mean difference of 2,6 and 2,5 log2 fold change, respectively, when compared to the shNT control cells (Fig 3C and 3D). This demonstrated that SNRNP200 plays a distinct role in the early and the late antiviral response pathways. The relationship between the affected gene subsets was assessed by resolving their interaction and functional alignment networks. The assessment showed that the top GO term for genes exclusively affected by SeV is “response to virus”, and the top GO term for IFN-α is “response to type I interferon”. Furthermore, it showed that SeV-specific genes affected by silencing SNRNP200 are IRF3-dependent (IFNB1, IL29, and BIRC6) and that several IFN-α-specific genes are JAK-STAT1-dependent (ACVR1C, CIQA, IFIT5, and OAS1). The latter might be explained by the reduction of IFN-induced *IRF9 and STAT1* mRNA levels encoding key transcription factors of ISGF3 that mediates signaling of type I and III IFNs (S2 Table). The molecular signature of differential gene expression strengthens the observation that SNRNP200 silencing hinders IRF3-dependent gene induction, leading to a general weakening of the RLR signaling pathway, and further suggests that SNRNP200 plays a distinct regulatory role in type I IFN signaling. The results suggest that SNRNP200 specifically regulates IRF3 activation upon RNA virus infection to promote IFNB1 induction and IFN effector responses, and thus demonstrates its importance in controlling viral infections.

### Sec63-1 domain of SNRNP200 is required for virus-mediated IFNB1 production

**Fig 4:**
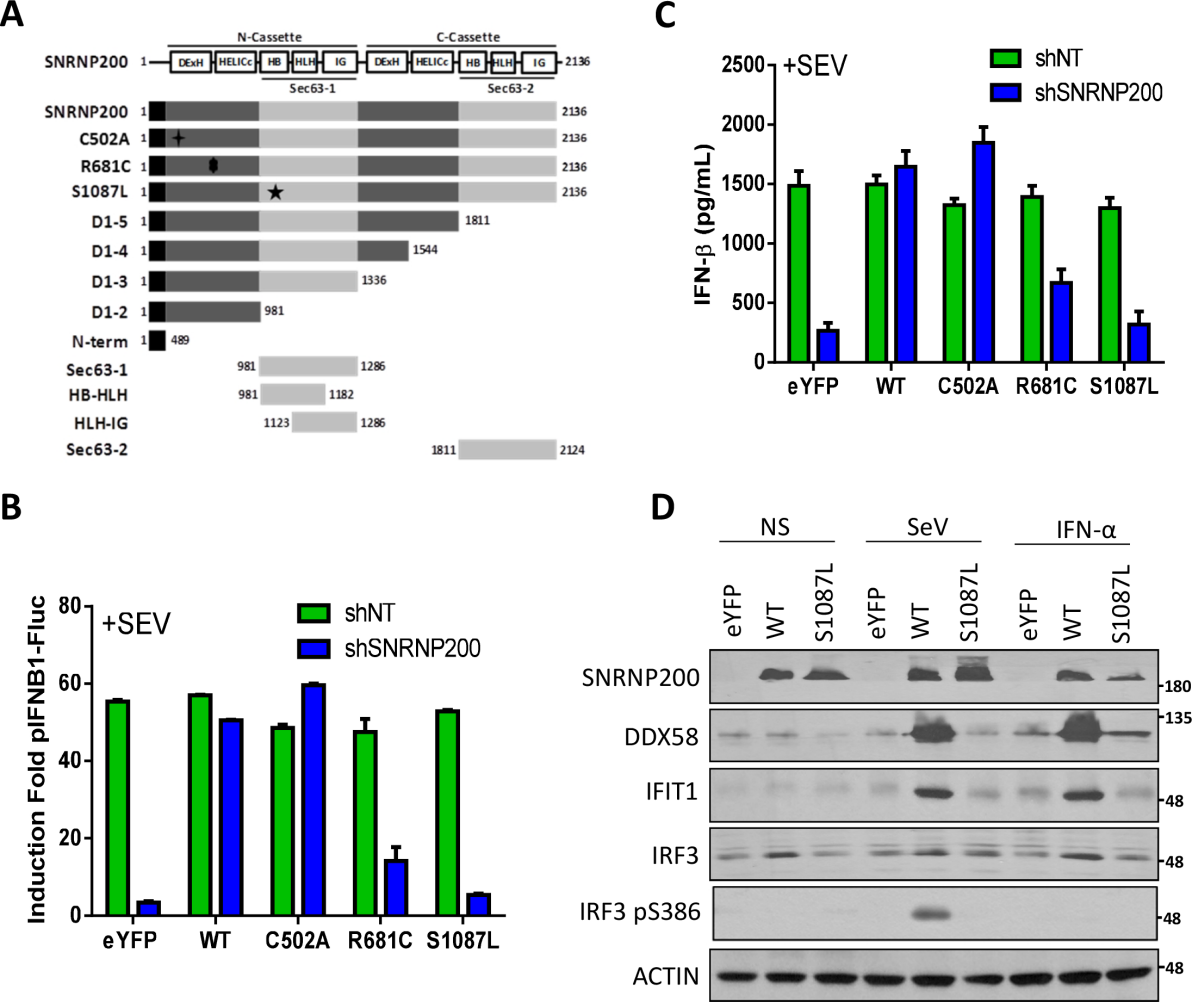
SNRNP200, but not Sec63-containing S1087L mutant, rescues SeV- and IFN-α-mediated induction of antiviral response in SNRNP200 KD cells. (A) Schematic representation of SNRNP200 protein, C-terminal truncation and clinically relevant mutants. (B) HEK 293T pIFNB1-Luc cells are transduced with shSNRNP200 and transfected with RNAi-resistant SNRNP200 WT and variants expression plasmids bearing the indicated mutation or eYFP as a control. Following 24 hours of SeV infection, total luciferase levels are measured and compared with control shNT cells. (C) HEK 293T are treated as indicated in B. IFN-β secretion levels are measured by ELISA and compared with shNT cells. (D) HEK 293T cells are transduced with shSNRNP200 and transfected with RNAi-resistant SNRNP200 WT and variants expression plasmids bearing the indicated mutation or eYFP as a control. At 24 hours, cells are harvested and DDX58, IFIT1, IRF3 and IRF3pS386 levels are resolved by immunobloting analysis of cell lysates.

To examine the manner in which SNRNP200 directly contributes to IRF3-mediated IFNB1 activation upon SeV infection, a series of recombinant SNRNP200-truncated mutants were tested for their ability to rescue IFNB1 reporter activity and ISG expression in SNRNP200 KD cells (Fig 4 and S9 Fig). None of the truncated mutants could induce an antiviral response in SNRNP200 KD cells (with the exception of a weak IFNB1 activation by expression of a D1-D3 construct). Indeed, the deletion of the C-terminal Sec63 domain (Sec63-2) alone, which was reported in yeast (Sec63-2 deleted Brr2 protein) to reduce ATPase/helicase activity and splicing [25], completely abolished the activation of IFNB1 promoter-driven reporter activity and the induction of IFIT1 and DDX58 upon SeV infection. To further explore a dual regulatory role in splicing and RNA-mediated antiviral responses, the described SNRNP200 heterozygous mutations associated with the autosomal dominant RP33 disease were considered [26–28]. In particular, the SNRNP200 S1087L and R681C variants located within the Sec63-1 homology domain and the N-terminal RecA-like ATPase/helicase domains, respectively, were investigated (Fig 4A). It was first demonstrated that the ectopic expression of RNAi-resistant WT SNRNP200 rescues the SeV-mediated IFN-β secretion and the IFNB1-driven reporter activity in KD cells, further validating the specificity and minimal off-target effects of shSNRNP200 and its associated immunoregulatory phenotype (Fig 4). Surprisingly, expression of the SNRNP200 S1087L mutant completely eliminated the ability to rescue IFNB1 activation (Fig 4B and 4C). Similar results were obtained using qRT-PCR as the rescue of endogenous IFNB1 mRNA levels was achieved only by the expression of WT SNRNP200 (S10 Fig). Concordantly, WT SNRNP200, but not the S1087L mutant, restores IRF3 protein levels, and more importantly, restores the phosphorylation of IRF3 at serine 386 as well as the inducible levels of DDX58 and IFIT1 upon SeV infection (Fig 4D). WT SNRNP200, but not the S1087L mutant, also restores IFN-α-dependent DDX58 and IFIT1 induction. It was also determined that expression of R681C variant only slightly rescues IFNB1 promoter-driven reporter activity and IFN-β secretion (Fig 4B and 4C). Interestingly, while investigating a mutation within the ATP binding motif, it was found that the ectopic expression of a SNRNP200 C502A variant elicited an IFNB1 response independent of viral infection (S11 Fig) in line with the recently reported natural gain-of-function of DDX58 and IFIH1 ATPase-deficient variants [29]. The constitutive induction of IFNB1 with expression of SNRNP200 C502A is further enhanced upon SeV infection to levels similar to the WT enzyme (Fig 4B and 4C), thereby suggesting the requirement of a functional SNRNP200 ATPase in conferring specificity to viral RNA and in preventing signaling through the recognition of self-RNA. Thus, the data firmly establishes a critical role of the Sec63-1 domain to promote virus-mediated IFNB1 production and further suggests a contribution of the N-terminal ATPase/helicase domain in sensing viral RNA.

### Sec63-1 domain of SNRNP200 is a major determinant of viral RNA recognition

**Fig 5:**
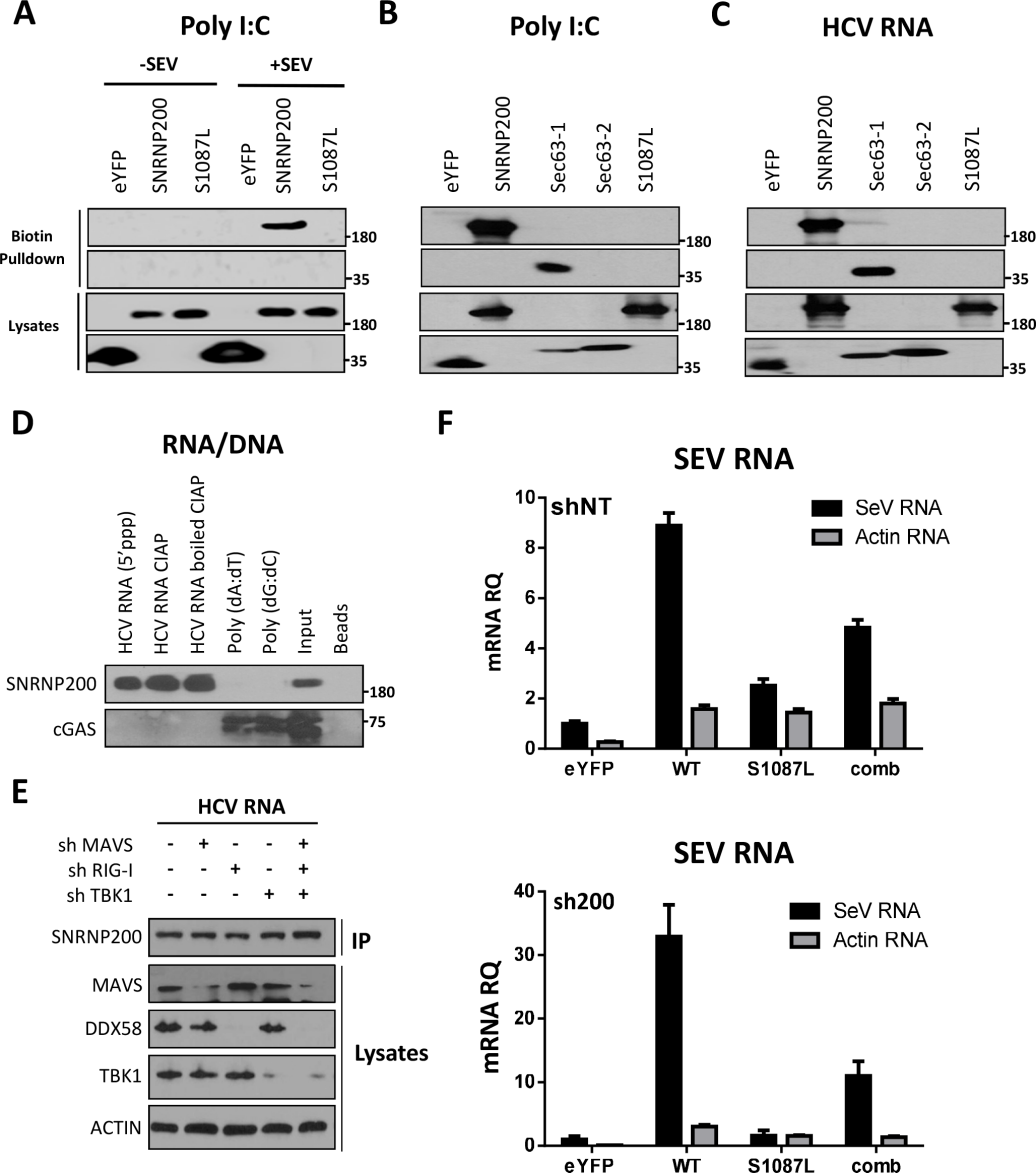
SNRNP200, but not S1087L mutant, binds viral RNA *in vitro*. (A) HEK 293T cells are transfected with FLAG-eYFP (control), FLAG-SNRNP200 or FLAG-SNRNP200 S1087L mutant expression plasmids for 48 hours and infected with SeV for 16 hours. RNA pull-down assays are performed with cell lysates using biotinylated poly (I:C). Cell lysates and bead-bound complexes are analyzed by Western blotting and compared to uninfected control cells. (B) HEK 293T cells are transfected with FLAG-Sec63-1 and FLAG-Sec63-2. RNA pull-down assays are performed and analyzed as indicated in (A). (C) HEK 293T are treated as indicated in (B) and RNA pull-down assays are performed on cell lysates using biotinylated HCV RNA and analyzed as indicated in (A). (D) HEK 293T cells are transfected with FLAG-SNRNP200 (top panel) or FLAG-cGAS (bottom) expression plasmids for 48 hours. RNA pull-down assays are performed with cell lysates using biotinylated HCV RNA (5’ppp) that is either left untreated or treated with CIAP or heat inactivated (h/i) CIAP (control), and with biotinylated poly (dA:dT) and poly (dG:dC) DNA molecules. Pull-down complexes are resolved by immunobloting and compared to protein input and uncoated beads as negative control. (E) Pull-down assays with biotinylated HCV RNA and transfected FLAG-SNRNP200 are performed with lysates of cells transduced with shRNA targeting MAVS, RIG-I or TBK1, either alone or in combination. (F) HEK 293T cells are transduced with shNT or shSNRNP200 and transfected with FLAG-eYFP, RNAi-resistant FLAG-SNRNP200 WT or S1087L variant expression plasmids and both (comb) for 48 hours. At 16 hours post-infection with SeV, cell lysates are subjected to an anti-FLAG immunoprecipitation, and RNA molecules are extracted from the immune complexes and analyzed by qRT-PCR. SeV and actin RNA levels are determined and normalized to RNA levels of cell lysates.

DExD/H-box helicases, such as RIG-I, are engaged in antiviral innate immunity because they detect viral nucleic acids and prevent the recognition of self-RNA through ATP hydrolysis (29). As the Sec63-1 containing S1087L mutation was reported to diminish binding to RNA duplex and to reduce RNA-stimulated ATPase/helicase activity without any discernible effect on the folding of SNRNP200 (27), it was hypothesized that this natural loss-of-function mutation abolishes the recognition of viral RNA for IFNB1 induction. To determine whether or not the S1087L variant impaired the binding of the immunostimulatory RNA in SeV-infected cells, the in vitro ability of exogenously expressed SNRNP200 to bind biotinylated polyinosinic-polycytidylic acid (poly (I:C)) was measured using an RNA pull-down and western blot analysis of bead-bound protein fractions (Fig 5). It was shown that FLAG-WT SNRNP200 binds poly (I:C), which is used as a viral double-stranded RNA (dsRNA) surrogate, only in SeV-infected cell extracts (Fig 5A). Furthermore, a complete loss of poly (I:C) binding by the FLAG-SNRNP200 S1087L variant was observed. Interestingly, the FLAG-Sec63-1 domain, but not the FLAG-Sec63-2, is sufficient to bind poly (I:C) (Fig 5B). These observations were confirmed with biotinylated HCV genomic RNA. As expected, WT SNRNP200 and the Sec63-1 domain, but neither the S1087L variant nor the Sec63-2 domain, were able to successfully pull-down HCV RNA (Fig 5C). To provide insight into the interaction of SNRNP200 and immunostimulatory RNA molecules, SNRNP200’s ability to bind a synthetic 5’-triphosphate (5’ppp) and a double-stranded stretch of RNA using the full-length HCV genome, produced in vitro by transcription with a T7 polymerase, was investigated (Fig 5D). A comparable binding of FLAG-WT SNRNP200 to the untreated and to the calf-intestine alkaline phosphatase (CIAP)-treated blunt-ended HCV RNA was observed. This comparable binding suggests that the 5’ppp moiety is not essential for the recognition of viral dsRNA. FLAG-SNRNP200 does not bind dsDNA molecules unlike the FLAG-cGAS control which does [30]. This is reflected by the lack of pull-down by FLAG-SNRNP200 with biotinylated polydeoxyadenylic acid-polythymidylic acid (poly (dA:dT)) and polydeoxyguanylic acid-polydeoxycytidylic acid (poly (dG:dC)) homopolymer molecules (Fig 5D). To assess the requirement of a protein complex with DDX58, MAVS, or TBK1 for binding HCV RNA, expressions of these proteins both individually and together were silenced, and RNA pull-down assays were performed to detect SNRNP200 (Fig 5E). It was determined that SNRNP200 binds HCV RNA regardless, ruling out a contribution of these proteins in its ability to recognize viral RNA. Finally, FLAG-tagged WT SNRNP200 and S1087L variant were immunoprecipitated upon SeV infection, and the co-purified RNA molecules were analyzed using qRT-PCR in SNRNP200 KD and in control shNT cells (Fig 5F). Increased amounts of actin mRNA for both immunoprecipitated proteins were found compared to the eYFP control (normalized to RNA levels of cell lysates). A significant enrichment of SeV RNA, which is more important in SNRNP200 KD cells than in shNT control cells that express the endogenous untagged protein, was observed upon immunoprecipitation of FLAG-WT SNRNP200, demonstrating a direct binding to viral genomes. The amount of SeV RNA recovered with the WT was almost 10- to 20-fold higher than with the S1087L variant in KD cells (and 3-fold in shNT cells), reflecting an altered RNA binding ability of the mutant. Despite the weak binding of SeV RNA by S1087L which is possibly due to its N-terminal RecA domains, the loss-of-function in IFNB1 induction reveals that this interaction is not biologically active. The data demonstrate that the Sec63-1 domain of SNRNP200 is a major determinant of viral RNA recognition and consequently of SNRNP200’s ability to activate antiviral innate immune responses.

### Sec63-1 domain of SNRNP200 interacts with TBK1

**Fig 6:**
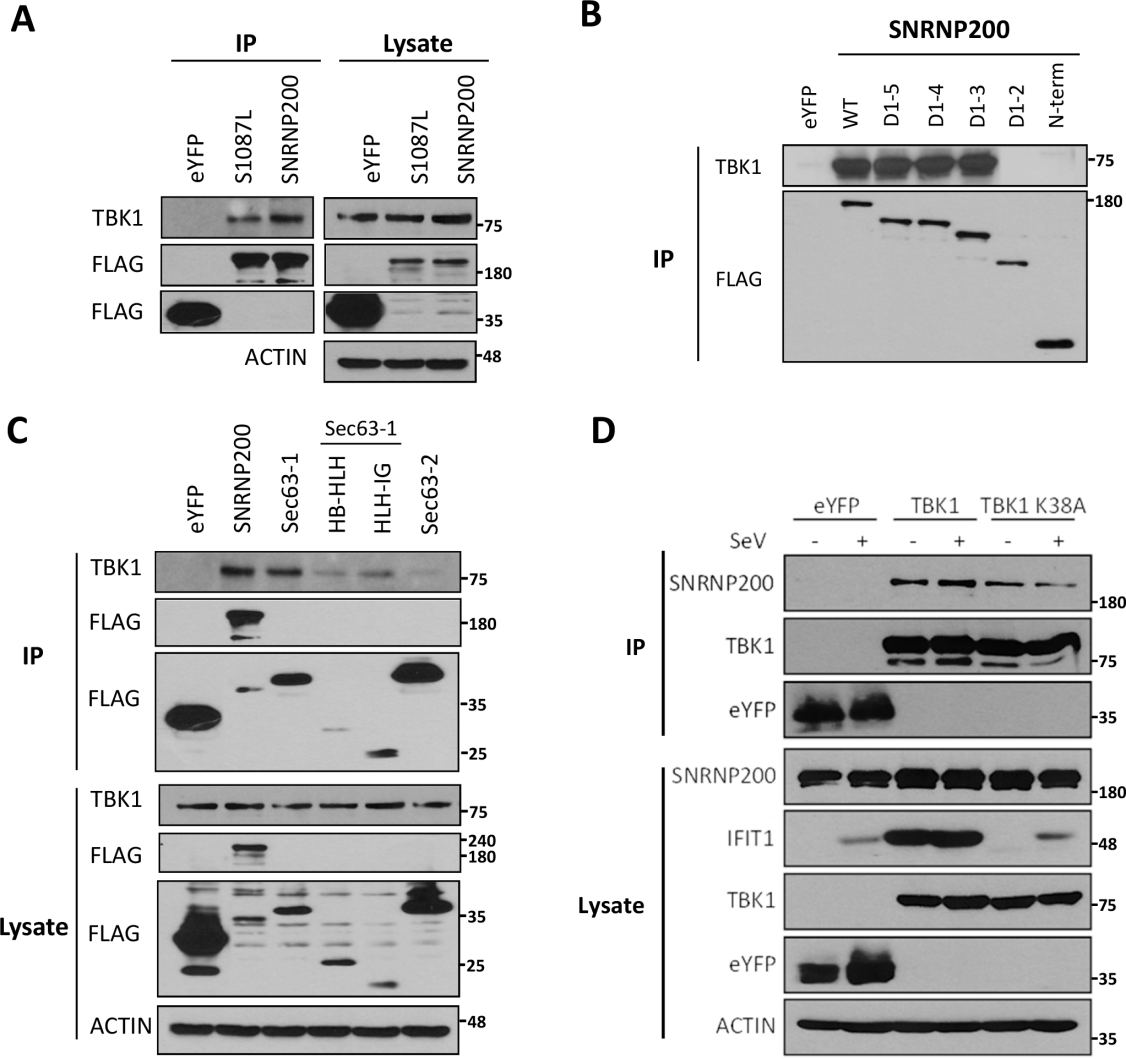
SNRNP200 Sec63-1 domain interacts with TBK1. (A) HEK 293T cells are transfected with FLAG-eYFP (control), FLAG-SNRNP200 or FLAG-SNRNP200 S1087L mutant expressing plasmids for 48 hours. Cell lysates are prepared following 16 hours of SeV infection and subjected to immunoprecipitation with anti-FLAG antibodies. Cell lysates and immune complexes are resolved by immunobloting analysis using anti-FLAG and anti-TBK1 antibodies. (B) Immunoprecipitation of FLAG-SNRNP200 C-terminal deletion mutants are performed and analyzed as indicated in (A). C) Immunoprecipitation of FLAG-SNRNP200 Sec63-1, HB-HLH or HLH-IG subdomains of Sec63-1 and Sec63-2 are performed and analyzed as indicated in (A). (D) Reciprocal immunoprecipitation of FLAG-eYFP (control), FLAG-TBK1 or FLAG-TBK1 K38A mutant following ectopic expression of SNRNP200 are performed as indicated in (A) and analyzed as indicated in (A). Cell lysates and immune complexes are resolved by immunobloting analysis using anti-FLAG and anti-SNRNP200 antibodies.

To better define a specific immunoregulatory role of SNRNP200, binding partners were identified by screening proteins of the antiviral signaling pathways upon immunoprecipitation of FLAG-tagged SNRNP200. This method successfully allowed the detection of a constitutive interaction between SNRNP200 and the ubiquitously expressed kinase TBK1. This interaction was also detected when using the SNRNP200 S1087L mutant (Fig 6A). The ability of various SNRNP200-truncated mutants (Fig 4A) to bind TBK1 (Fig 6B) was then assessed. A mutagenesis analysis showed that the Sec63-1 domain of SNRNP200 is required and sufficient for TBK1 interaction (Fig 6C), which is similar to the observation for RNA binding (Fig 5B and 5C). Both Sec63 homology domains of SNRNP200 contain a helical bundle (HB) and immunoglobulin-like (IG) sub-domains separated by a helix loop helix (HLH) motif. To more accurately map the TBK1 binding domain, the sub-domains of Sec63-1 were expressed separately. A weak interaction with the HLH-IG sub-domain was observed, suggesting its contribution to the binding of TBK1 (Fig 6C). It was also demonstrated that the C-terminal Sec63-2 domain could not bind TBK1, which corroborates the detected interaction of the N-terminal truncated D1-3, D1-4, and D1-5 mutants with TBK1 (Fig 6B and 6C). In reciprocal experiments, immunoprecipitation of FLAG-tagged TBK1 confirmed the interaction with ectopically expressed SNRNP200 in uninfected and in SeV-infected cells (Fig 6D). In addition, the kinase-dead TBK1 mutant (K38A) was still shown to interact with SNRNP200, demonstrating that this interaction is not dependent on TBK1 activity (Fig 6D). Finally, the interaction was further confirmed in A549 cells by the co-immunoprecipitation of endogenous SNRNP200 and TBK1 proteins (S12 Fig).

**Fig 7:**
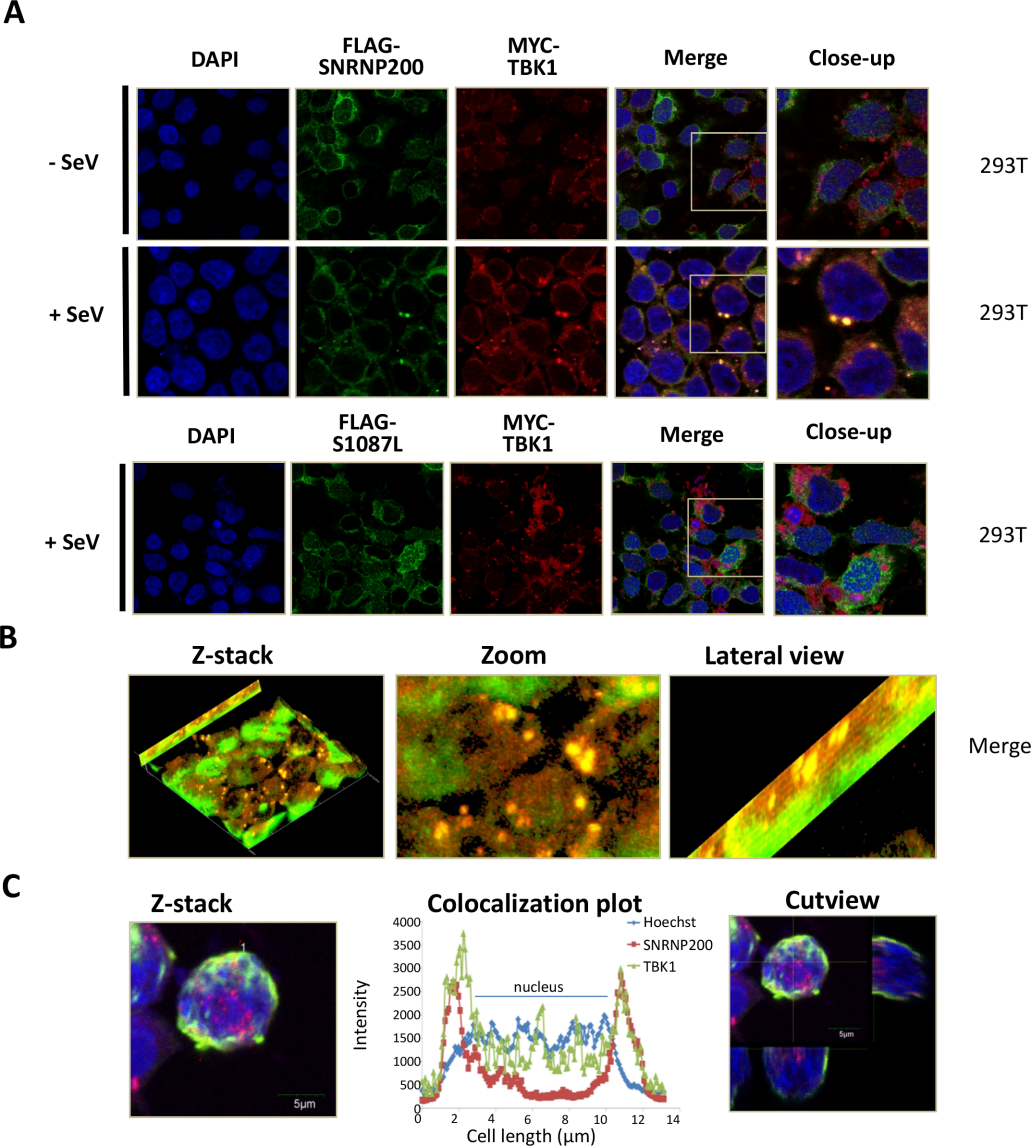
Re-localization of SNRNP200 in perinuclear cytoplasmic speckles and colocalization with TBK1 in response to SeV infection. (A) Confocal analysis of HEK 293T co-transfected with FLAG-SNRNP200 or FLAG-SNRNP200-S1087L and MYC-TBK1 using Hoechst, anti-FLAG and anti-MYC antibodies without virus infection or following a 16-hour infection with SeV. Imaging was done using a 63x/1.40 Oil DIC objective. Intensity analysis showed that 19/19 cells have cytoplasmic colocalization between SNRNP200 and TBK1 in SeV-infected cells. (B) Z-stack and lateral view of SNRNP200 and TBK1 in SeV-infected HEK 293T cells treated as indicated in (A). (C) Z-stacks reconstitution of a complete cell with colocalization plot and cut view, showing an exclusive cytoplasmic colocalization of SNRNP200 and TBK1.

To further assess the interaction of SNRNP200 and TBK1, their intracellular localization was investigated by examining confocal fluorescence microscope images of HEK 293T cells and of HeLa cells in response to SeV infection (Fig 7 and S13 Fig). It was observed that FLAG-SNRNP200 (HEK 293T cells) and endogenous SNRNP200 (Hela cells) are localized to the nucleus and cytoplasm with a diffuse staining prior to stimulation. Upon viral infection, a subcellular fraction of SNRNP200 relocalizes with TBK1 into perinuclear cytoplasmic speckles (Fig 7A and S13B Fig). SNRNP200 and TBK1 colocalization can be easily observed in the 3D-stack and lateral view of infected cells (Fig 7B and 7C). Unlike WT SNRNP200, the staining of the FLAG-SNRNP200 S1087L mutant shows neither relocalization of the protein nor colocalization with TBK1 into these cytoplasmic speckles upon infection (Fig 7A and S13A Fig), correlating with its lack of RNA binding (Fig 5). Thus, the data suggest that viral RNA recognition by the Sec63-1 domain is responsible for the relocalization of SNRNP200 to perinuclear cytoplasmic speckles, and that SNRNP200 possibly functions as a novel adaptor via its interaction with TBK1 to promote IRF3 phosphorylation and antiviral innate responses.

### SNRNP200 regulates innate immune responses of SeV-infected human MDM

**Fig 8:**
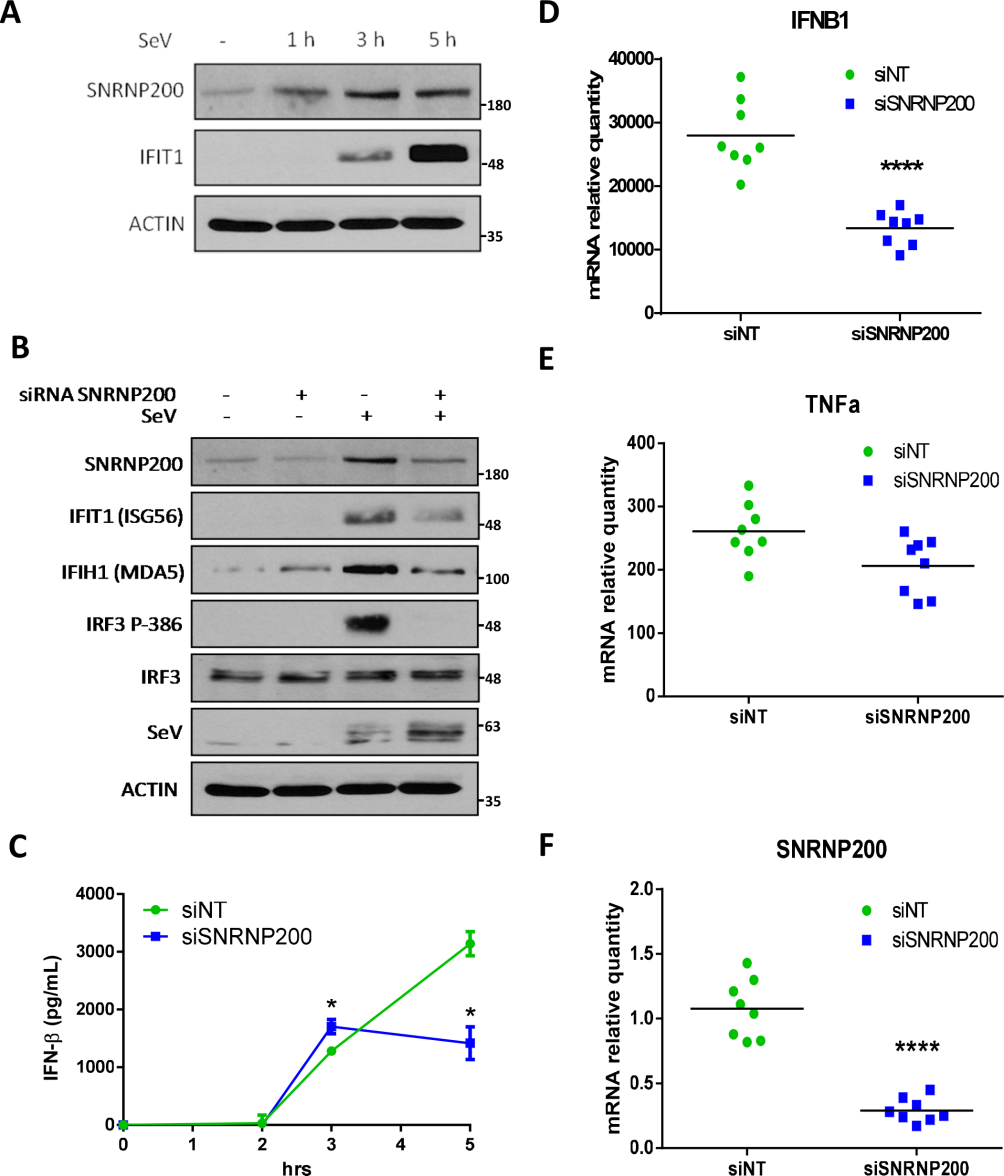
SNRNP200 KD restricts SeV-mediated antiviral response of human MDM. (A) MDM are infected with SeV for 1, 3 or 5 hours. SNRNP200 and IFIT1 protein levels are resolved by immunobloting of cell lysates. (B) MDM are transfected with a pool of siRNA targeting SNRNP200 for 48 hours and infected with SeV. At 3 hours post-infection, cells are harvested and selected proteins (SNRNP200, IFIT1, IFIH1, IRF3, IFR3-386, SeV and actin) are resolved by immunobloting of cells lysates and compared to control cells treated with scrambled siRNA. (C) MDM are transfected with a pool of siRNA targeting SNRNP200 for 48 hours and infected with SeV for 1, 3 or 5 hours. Supernatants are harvested and IFN-β secretion levels are measured by ELISA and compared to control cells treated with scrambled siRNA (siNT). (D-F) MDM are transfected with a pool of siRNA targeting SNRNP200 (siSNRNP200) or scrambled siRNA (siNT) and infected with SeV for 1 hour. Cells are harvested and relative gene expression of IFNB1 (D), TNFα (E) and SNRNP200 (F) are measured by qRT-PCR and compared with scrambled control cells. mRNA RQ are normalized versus *ACTIN* and *HPRT1* mRNA. P values <0.0001 (****) are indicated. Data are pooled results from two experiments of two biological replicates.

The regulation of antiviral responses by SNRNP200 was further investigated in immune cells using primary cultures of purified human monocyte-derived macrophages (MDM). It was found that SeV infection leads to an increase in the immunodetection of SNRNP200 without affecting mRNA levels (Fig 8A), as observed in SeV-infected and in IFN-α-treated HEK 293T cells (S14 Fig). More importantly, the silencing of SNRNP200 in MDM decreases the induction of IFIH1 and IFIT1, and completely blocks IRF3 Ser386 phosphorylation within 3 hours post-infection (Fig 8B). Kinetic studies on IFN-β production have further demonstrated a complete blockage of its secretion at 3 hours post-infection (Fig 8C). In contrast to the unchanged TNFα mRNA levels, a decrease in IFNB1 mRNA was observed, which correlates with the reduced SNRNP200 mRNA at 1 hour post-infection in MDM (Fig 8D, 8E and 8F). Interestingly, the duration of SNRNP200 gene silencing is not sufficient to affect the steady-state levels of IRF3 protein, though it completely inhibits its phosphorylation. In addition, SNRNP200 KD increased SeV protein levels, as observed in HEK 293T cells (Figs 1C and 8B). These results confirm a regulatory role of SNRNP200 in the IRF3-mediated antiviral response in human macrophages.

### Impaired antiviral response of PBMCs from RP33 patients

**Fig 9:**
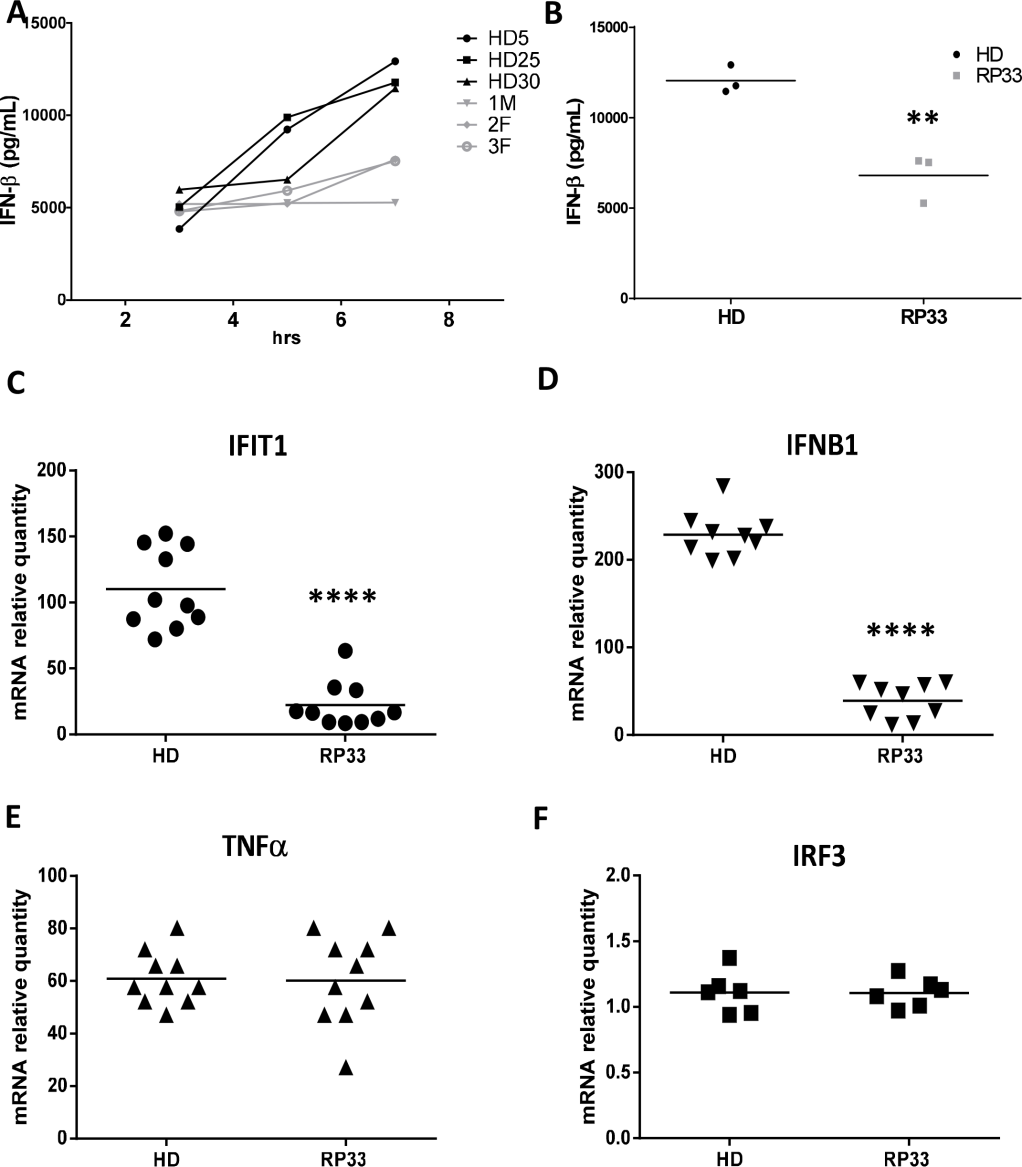
PBMCs of RP33 patients bearing monoallelic point mutation in SNRNP200 show hindered antiviral innate immune response. (A) PBMCs of RP33 patients are infected for 3, 5 and 7 hours with SeV. Supernatants are harvested and IFN-β secretion levels are measured by ELISA and compared to PBMCs of three healthy donors (HD). (B) Alternative representation of the 7-hours SeV infection of individual RP33 patients and HD as in (A), where the horizontal bar represents the mean of each group. P value <0.01(**) is indicated. (C-F) PBMCs of RP33 patients are infected for 1 hour with SeV. Cells are harvested and relative gene expression of IFIT1(C), IFNB1 (D), TNFα (E) and IRF3 (F) are measured by qRT-PCR and compared with PBMCs of HD. mRNA RQ are normalized versus *ACTIN* and *HPRT1* mRNA. P values <0.0001 (****) are indicated.

RP is an inherited degenerative eye disease that causes severe vision impairment and blindness due to mutations in several core spliceosomal proteins. The antiviral responses of peripheral blood mononuclear cells (PBMCs) of RP33 patients that are genotyped for a particular monoallelic mutation in SNRNP200 were characterized: p.S1087L- c.3260C>T in the Sec63-1 domain and p.R681C c.2122G>A in the N-terminal helicase domain (see S1 Table for donor information). Interestingly, all RP33 patients showed a complete blockage of IFN-β cytokine production at 3 hours post-infection with a significant two-fold reduction in IFN-β secretion at 7 hours (Fig 9A and 9B). The decreased IFN-β production was corroborated by the reduction of virus-induced IRF3-dependent IFNB1 and IFIT1 mRNA. On the other hand, NF-κB-dependent TNF mRNA levels were not significantly affected (Fig 9C, 9D and 9E). The IRF3 mRNA levels determined by qRT-PCR showed no difference between healthy donors (HD) and RP33 patients (Fig 9F). Finally, a cytokine 41-plex assay performed on supernatants of infected PBMCs from HD and RP33 patients showed a significant decrease in IFN-α2, but showed similar cytokine/chemokine levels of RANTES, IL6, CXCL10, and IL1B (S15 Fig). The defective antiviral response of PBMCs from RP33 patients demonstrates that SNRNP200 plays a crucial role in regulating the IRF3-dependent pathway of IFNB1 production and does so without altering the NF-κB-dependent inflammatory pathway.

## Discussion

**Fig 10:**
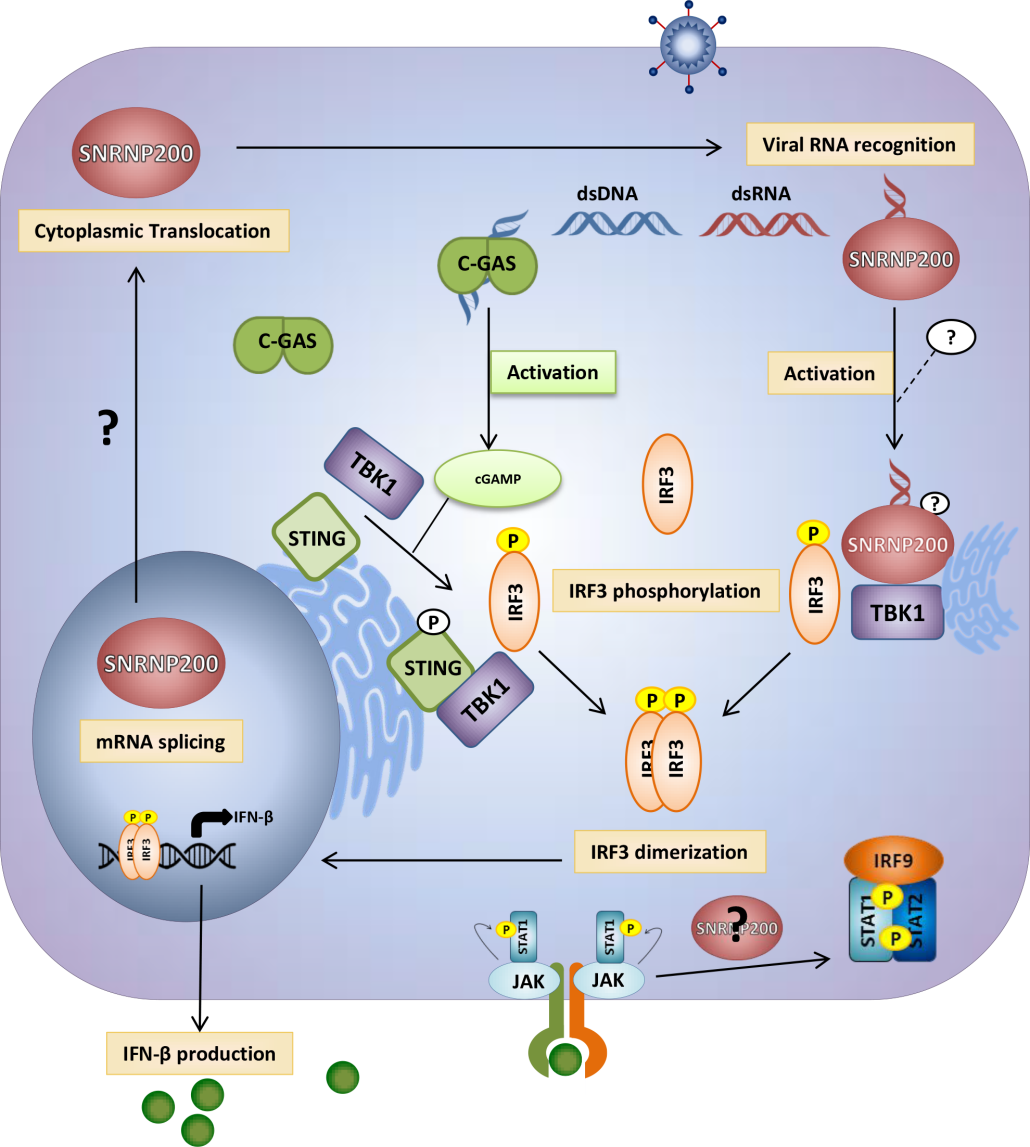
SNRNP200 regulates the antiviral response. Upon viral infection, SNRNP200 relocates to an undefined cytoplasmic structure where it is able to directly sense viral RNA via its Sec63-1 domain. This activation by viral nucleic acids, through an undefined mechanism, results in a virus-induced association of SNRNP200 with TBK1 into a larger order perinuclear structure. The mobilization of SNRNP200 with TBK1, downstream of DDX58/MAVS signaling, promotes IRF3 phosphorylation and IRF3’s subsequent translocation to the nucleus. This nuclear translocation allows the transactivation of the IFN-β promoter and thus the production of type I IFNs and ultimately of ISGs. This model demonstrates that SNRNP200 is dispensable to cGAS/STING cytosolic DNA sensing but required for RLR/MAVS/TBK1/IRF3 signaling, by a novel mechanism, to engage antiviral immunity against RNA viruses.

SNRNP200 RNA helicase is ubiquitously expressed in cells and is a core component of the spliceosome. Its plays a key role in unwinding U4/U6 snRNA to form a highly structured RNA interaction network among the U2, U6, and U5 snRNA and the pre-mRNA required for activation of the spliceosome [31,32]. Despite this critical function for pre-RNA splicing, to the authors’ knowledge, no data has been found that suggests a role of SNRNP200 in host defense. Furthermore, few studies have described a contribution of spliceosomal proteins for innate immunity. Two spliceosomal proteins (SRSF1 and SF3A1) have been identified from the genome-wide gene silencing screening (Fig 1A) that have previously been reported to be involved in the generation of alternative splice variants of important innate immune regulators. Depletion of SRSF1 in human A549 lung cancer cells reduces IFN-β through the expression of alternative IRF3 spliced variants [10], while SF3A1 silencing leads to a decreased induction of pro-inflammatory cytokines by promoting an alternative splice form of MyD88 [11]. Based on this study, evidence of a novel role of the spliceosomal SNRNP200 RNA helicase in the regulation of IRF3-mediated antiviral response upon the RNA virus infection in human cells is presented: 1. SNRNP200 KD cells infected with SeV and FLUA show higher virus titers and viral proteins (Fig 1 and S2 Fig), suggesting that SNRNP200 is involved in host defense mechanisms; 2. SNRNP200 KD cells reduce virus-mediated IFN-β production (Figs 1B, 2E, 4C and 8C); 3. Epistasis studies suggest a role for SNRNP200 within the antiviral response during IRF3 activation (Figs 1E, 1F, 2B, 2D and 2E); 4. SNRNP200 solely regulates the RLR pathway and does not affect the TRIF or the cGAS/STING pathways when activating IFNs production (Fig 2B, 2D, 2E and S4 Fig); 5. SNRNP200 requires a competent Sec63-1 domain and functional ATPase/helicase activity to promote IRF3-dependent IFNB1 activation (Fig 4); 6. The SNRNP200 Sec63-1 domain binds immunostimulatory RNA molecules (Fig 5); 7. SNRNP200 interacts with endogenous TBK1 through its Sec63-1 domain (Fig 6); and 8. PBMCs of RP33 patients (who have one allele carrying the dominant S1087L or R681C mutation) showed a reduction of IFN-β secretion when challenged with SeV (Fig 9). Thus, SNRNP200, the only Ski2-like RNA helicase involved in pre-mRNA splicing, regulates IRF3-dependent IFNB1 production upon RNA virus infection through the recognition of viral RNA promoting the phosphorylation of IRF3 and possibly functions as an adaptor protein through its constitutive interaction with TBK1. The results reveal a unique molecular mechanism regarding the way SNRNP200 regulates the antiviral response. Mechanistically, it was found that, upon viral infection, SNRNP200 relocates to some undefined cytoplasmic structures were it is able to directly sense viral RNA. This activation results in a striking virus-induced association of SNRNP200 and TBK1 into larger order punctate perinuclear structures. This mobilization of SNRNP200 and TBK1 promotes IRF3 phosphorylation that, in turn, translocate to the nucleus to transactivate the IFN-β promoter and induce the production of ISGs to fully engage antiviral immunity (for a model, see Fig 10).

The major mechanism by which SNRNP200 functions is as a spliceosomal helicase that unwinds the U4/U6 snRNA, providing key remodeling activity for spliceosome catalytic activation, and thus it regulates the expression of a large and disparate group of genes associated with the cell cycle [33]. Indeed, the transcriptional profiles of HEK 293T cells indicate a large group of differentially expressed genes upon SNRNP200 silencing that are associated with the immune system and the cell cycle, as shown by the Reactome Pathway Enrichment Analysis (S8A Fig). Nevertheless, among the total SeV- and IFN-induced genes of control shNT cells, silencing SNRNP200 KD affects specific SeV- and IFN-inducible genes as well as common genes by more than 1,5 log2 fold induction (see Venn diagrams of S8 Fig). The analysis of the gene network confirmed an enrichment for innate immunity gene function (Fig 3E) and revealed that altered genes (52 for SeV, 55 for IFN, and of which 13 are common to both) are highly connected to IRF3 and IFNB1 by a molecular signature, which indicates that SNRNP200 silencing hinders IRF3-dependent gene induction. One possible mechanism is that SNRNP200 affects the pathway at a transcriptional level, as first revealed by the observation that SNRNP200 alters expression of the key transcription factor IRF3, which is essential for *IFNB1* transcription. The decrease of IRF3 mRNA and protein levels correlates with the reduced SNRNP200 mRNA and protein levels as well as with the reduced expression of effector genes upon infection in KD cells (S5 Fig); however, the experiments did not identify splicing variants to explain the reduced IRF3 protein levels (S7 Fig), ruling out an alternative splicing regulation of *IRF3* mRNA for SNRNP200 depleted cells. While the reduced expression of IRF3 and DDX58 proteins (S5A Fig) may contribute to the phenotype, considerable evidence suggests that it is not the primary mechanism responsible for the decreased IFNB1 activation of SNRNP200 KD cells. First, ectopic expression of IRF3 and/or DDX58 fails to restore the virus-mediated IFNB1 production or IFIT1 expression in SNRNP200 KD cells, while expression of the constitutively active IRF3 (5D) fully rescues the antiviral response (Figs 1F, 2B, 2D, 2E and S6 Fig). Second, activation of the cGAS/STING pathway involved in the recognition of cytosolic DNA is not affected by SNRNP200 KD, despite the reduced IRF3 protein levels (Fig 2C) which slightly restricts IFNB1 production when cGAS/STING activation and SeV infection are combined (Fig 2D and 2E, see cGAS+STING versus cGAS+STING+IRF3). Indeed, the full activation of the cGAS/STING/TBK1/IRF3 pathway in SNRNP200 KD cells further supports a specific role for SNRNP200 upon activation of the RLR/MAVS/TBK1/IRF3 pathway by RNA virus infection. Finally, the silencing of SNRNP200 completely blocks IRF3 Ser386 phosphorylation in MDM, even when IRF3 protein levels are similar to control cells, resulting in the blockage of IFN-β secretion at 3 hours post-infection (Fig 8C). On the other hand, gene profiling data clearly illustrated that SNRNP200 affects the expression of a large group of genes associated with the immune system and the cell cycle of unstimulated cells (S8A Fig). Furthermore, it was observed that silencing SNRNP200 reduces type I IFN signaling downstream of STAT1 phosphorylation through a molecular mechanism that requires further investigation. Thus, it cannot be ruled out that the perturbation of pre-mRNA processing leading to impaired expression of immune-related genes possibly contributes to the reduced antiviral response of SNRNP200 KD cells via a global transcriptional regulatory role. Nonetheless, the abrogated phosphorylation of IRF3 (Fig 2A and 2B for IRF3p386/IRF3 ratios and 8B) provides the first mechanistic insight to explain the phenotype of SNRNP200 depleted cells. Based on these results, a direct role of SNRNP200 was considered for the activation of IRF3 that leads to IFNB1 production. To determine the regulatory function of SNRNP200 in IRF3 phosphorylation, its ability to interact with known members of the RLR signaling pathway was evaluated and its interaction with TBK1 was carefully characterized. The TBK1 binding site to the Sec63-1 domain (Fig 6C) was mapped, and a colocalization of SNRNP200 and TBK1 in cytoplasmic speckles triggered by SeV infection was observed (Fig 7 and S12 Fig). This indicates the involvement of a cytoplasmic SNRNP200-TBK1 protein complex in the modulation of IRF3 phosphorylation required for IRF3’s dimerization and nuclear translocation for *IFNB1* transcription and for ISGs production [34] in a mechanism similar to that described for DDX3 helicase [19].

The hypothesis that SNRNP200 directly functions as a sensor of viral RNA was tested using RNA pull-down experiments. It was demonstrated that SNRNP200, and more specifically its Sec63-1 domain, binds viral surrogate poly (I:C) and HCV genomic RNA (Fig 5B and 5C). The RNA-binding ability of SNRNP200 mainly involves the recognition of dsRNA, as seen with poly (I:C), while the presence of the 5’triphosphate moiety as well as the expression of DDX58, MAVS, and TBK1 proteins are not required for binding dsRNA molecules (Fig 5D and 5E). As expected, SNRNP200 RNA helicase does not bind dsDNA (Fig 5D), which supports the observation that SNRNP200 KD does not affect ISG56 promoter-driven activity upon activation by the cGAS/STING pathway (S4B Fig). Surprisingly, in contrast to DDX58, which binds poly (I:C) in the absence of viral infection (S16 Fig), the binding of SNRNP200 to dsRNA was only observed following SeV infection. This observation cannot be explained, especially because SNRNP200 binds SeV RNA with high affinity, as reflected by the significant enrichment of the protein pull-down over cell lysates of SNRNP200 KD cells (with low SeV RNA levels detected due to the rescue of antiviral response by WT expression) (Fig 5F). It was further demonstrated that expression of the naturally occurring SNRNP200 S1087L variant located in the Sec63-1 domain, which is associated with RP33 [OMIM:610359], is unable to bind dsRNA and SeV RNA (Fig 5A and 5F), does not relocalize with TBK1 upon SeV infection (Fig 7A), and cannot restore the antiviral response in SNRNP200 KD cells (Fig 4D). Thus, the results suggest that a pre-activation of SNRNP200 upon recognition of viral RNA allows its relocalization to perinuclear cytoplasmic speckles with TBK1. Finally, the study of ATPase/helicase-deficient SNRNP200 variants that affect the antiviral response and the discovery of a mutant (C502A) that elicits an IFNB1 response independent of viral infection and fully rescues IFN-β in SNRNP200 KD cells (Fig 4C and S11 Fig) further support the SNRNP200 ATPase/helicase function in conferring specificity to viral RNA and preventing signaling through the recognition of self-RNA, as recently reported for the natural gain-of-function DDX58 and IFIH1 ATPase-deficient variants [29]. These data demonstrate a direct regulatory role of SNRNP200 via its Sec63-1 domain and its ATPase/helicase function in the recognition of viral RNA and in the relocalization into cytoplasmic speckles with TBK1 to promote IRF3 activation and the antiviral response.

To ascertain the regulatory role of SNRNP200 in immune cells, depletion experiments were carried out with purified MDM isolated from PBMCs of healthy donors. As observed with the cell lines, SNRNP200 KD led to a hindered IFN-β production by blocking IRF3 phosphorylation without altering IRF3 expression in MDM (Fig 8B and 8C). There was also an increased viral susceptibility, illustrating a relevant role of SNRNP200 in the antiviral response of human macrophages. Furthermore, the loss-of-function mutations of the human SNRNP200 gene that cause autosomal-dominant RP33 were exploited. RP is a rare inherited disease of retinal dystrophies with an incidence of one in 3,000–4,000, of which 1.6% bear mutations in the SNRNP200 gene [35]. The investigated S1087L is a disease-associated mutation with complete penetrance in the RP33-linked family [36–38]. With access to PBMCs of three RP33 patients who had one allele carrying the dominant S1087L or R681C mutation, a decreased IRF3-dependent antiviral response was confirmed, when challenged with SeV, by the specific reduction of IFN-β and IFN-α2 cytokine secretion, without any effect on other tested cytokines (Fig 9 and S15 Fig). Thus, further evidence with human cells of patients with RP33 showed that SNRNP200 positively regulates antiviral responses independently from its primary core function in pre-mRNA splicing.

The recent resolution of the SNRNP200 structure (amino acids 395 to 2129) provides the spatial relation between duplicated N-terminal and C-terminal cassettes that both contain a RecA1-RecA2 DEAD-box helicase domain and a Sec63 homology domain [27]. Both cassettes are required for optimal helicase activity and splicing function, but only the N-terminal cassette is reported to be catalytically active [27]. The 3D structure of the Sec63-1 homology region resembles the structure of isolated C-terminal Sec63 units in yeast and in human enzymes [25,39]. The 3D structure showed that the serine 1087 is located on a long scaffolding helix (referred as a ratchet-helix), within the HB domain. The HB domain forms the top, while the RecA domains form the bottom, of a central tunnel that act as a strand separation device for RNA. The testing of a leucine at position 1087 exhibited decreased RNA binding and reduced ATPase and helicase activities compared to the WT [27]. This mutation is believed to decrease spliceosome activation and to explain its linkage to RP33 [25]. In this study, the S1087L mutant completely abolished the recognition of poly (I:C) and of viral RNA molecules and was completely unable to relocalize into TBK1-containing cytoplasmic speckles, which could explain the hindered antiviral response. Further proteomic studies should provide a more comprehensive explanation for this mechanism along with the identification of interaction partners that mediate the cytoplasmic relocalization of a SNRNP200-TBK1 complex upon infection. Structurally, SNRNP200 also decisively differs from other spliceosomal helicases, as it belongs to the Ski2-like subfamily, which is a small family within the superfamily 2 helicases (founder member: yeast Ski2) involved in a variety of RNA processing and degradation events [40]. In addition to SNRNP200, which exhibits the ATP-dependent unwinding activity of U4/U6 RNA duplexes during pre-mRNA splicing [22,41,42], other helicases, such as SKIV2L and DDX60, promote exosome-mediated RNA decay [16,43]. SKIV2L is involved in the elimination of incompletely spliced RNA transcripts upon stress responses, which triggers a sterile RNA-activated antiviral innate response due to its inhibition [43]. Indeed, SKIV2L-deficient patients exhibit a constitutive type I IFN signature in their peripheral blood that results in a human auto-immune disorder. Moreover, the yeast Ski2 and Ski2-like helicase 1 (Slh1) have been implicated in antiviral defense by blocking the translation of RNA lacking a 3' poly(A) structure [44,45]. Further studies are required to assess the potential role of SNRNP200 for the recognition of host RNA molecules upon stress responses.

Finally, to the authors’ knowledge, there has been no association between RP33 pathology and immune disorders. The findings in the PBMC of patients with a monoallelic point mutation in SNRNP200 established a deregulation of innate immunity, which may affect cell viability in different retinal disorders, as these cells and neural cells are usually non-proliferative and long-lived. Although RP33 is a rare event, it may be clinically relevant in identifying the mechanism of disease onset at a molecular level in relation to a deregulation of the innate response and the control of cell viability. Indeed, mutation E50K of optineurin, a critical regulator of antiviral signaling [46], promotes interaction with TBK1 and is associated with familial primary open-angle glaucoma [47]. The dysfunction of optineurin and TBK1 in retinal cells is assumed to play a significant role in glaucomatous and other retinal diseases by affecting the autophagy process and survival [48,49].

In summary, it has been demonstrated that upon virus infection, the SNRNP200 RNA helicase in combination with TBK1 via its Sec63-1 domain recognizes viral RNA, relocalizes into TBK1-containing cytoplasmic structures, and positively regulates IRF3 phosphorylation to promote the antiviral response. The regulatory role of SNRNP200 is confirmed in the MDM and PBMCs of RP33 patients due to the impaired production of IFN-β upon viral infection. The data revealed a crucial immunoregulatory role for the SNRNP200 helicase as well as for the Sec63-1 domain within SNRNP200, which acts as an RNA sensor and as an adaptor for TBK1 to promote the IRF3-mediated antiviral innate immune response. Taken together, the data illustrate a novel function for SNRNP200 that is clearly distinguishable from its function in spliceosome activation and in pre-mRNA splicing. Through the development of immunomodulatory molecules, exploiting the function of human encoded regulators of the antiviral response presents an alternative strategy in treating a broad range of viral infections.

## Materials and Methods

### Ethics statement

This study was approved by Institutional Review Board (IRB) of the participating institution (McGill Children’s Hospital, McGill University Health Centre and Centre Hospitalier Universitaire de l’Université de Montréal (CHUM)) and written informed consent was obtained from all participants before participation.

### Expression vectors

*SF3A1*, *NHP2L1* and *PHF5A* cDNAs were purchased from GE Dharmacon/Open Biosystems. Following PCR-amplification, PCR products were cloned into pcDNA3.1-Hygro-MCS using EcoRV/HindIII[6]. SNRNP200 was subcloned from the pBluescriptSK-hBrr2 obtained from R. Lührmann [50] into pcDNA3.1-Hygro(+) (Life Technologies) using NotI and XhoI restriction sites. SNRNP200 deletion mutants and S1087L point mutation were generated by PCR. All constructs were verified by Sanger sequencing and subsequent western blot analysis. If necessary, validated constructs where subcloned into pcDNA3.1-MCS-FLAG. pIFNB1-LUC and p2xNF-κB-LUC luciferase reporter constructs were previously described [51–53]. Generation of stable HEK 293T cells harboring the pIFNB1-LUC and pEF1α-LUC promoters was previously described [8].

### Cells lines and culture

Human embryonic kidney HEK 293T (ATCC), human epithelial adenocarcinoma HELA (ATCC) and human hepatoma cell lines Huh7 / Huh7.5 (ATCC) were cultured in Dulbecco's modified Eagle's medium (DMEM, Wisent). Human lung adenocarcinoma epithelial A549 (ATCC) were cultured in Ham’s F-12 medium (Life Technologies). Both media were supplemented with 10% fetal bovine serum, 100 units/ml penicillin, 100 μg/ml streptomycin and 2 mM glutamine (all from Wisent) at 37°C in an atmosphere of 5% CO_2_. Transient transfections were performed with lipofectamine 2000 (Life Technologies) according to manufacturer’s protocol. Peripheral blood mononuclear cells (PBMCs) were isolated from fresh heparinized peripheral blood samples by Ficoll-Histopaque gradient centrifugation (Sigma-Aldrich). Unfrozen PBMCs were washed twice in 10 ml of sterile RPMI 1640 and re-suspended in RPMI 1640 supplemented with 10% FBS. PBMCs were counted using a haemocytometer and counts were adjusted using trypan blue exclusion to plate 1x10^6^ PBMCs in 100 μl RPMI 1640 supplemented with 10% FBS in 96-well plate. For monocyte-derived macrophage (MDM), PBMCs were harvested has described above and monocyte were isolated using MACS Monocyte Isolation Kit II human (Miltenyi Biotec) as per manufacturer’s protocol before differentiation into MDM for five days in the presence of 10 ng/mL granulocyte-monocyte colony stimulating factor (M-CSF, R&D).

### shRNA and siRNA gene silencing

shRNAs from MISSION TRC shRNA lentiviral library (Sigma-Aldrich) were used as followed: shRNA targeting SNRNP200 (TRCN0000051831), SF3A1 (TRCN0000006597), PHF5A (TRCN0000074878), NHP2L1 (TRCN0000074799), or shRNA non-target (NT). shRNA were transfected in combination with a standard packaging mix (1.5 μg pMDLg/pRRE, 1.5 μg pRSV-REV and 3 μg pVSVg) as previously described[54]. siRNA ON-TARGETplus SMARTpool, Human SNRNP200 and siRNA non-targeting #1 Human, ON-TARGETplus (GE Healthcare, Dharmacon), Santa Cruz HELIC2 siRNA (h) (sc-75243) were transfected with lipofectamine RNAi Max (Life Technologies) for 48 hours according to manufacturer’s protocol.

### Firefly luminescence assay

For assays in 96-well plates, cells were seeded in white 96-well plates at a density of 5,000 HEK 293T pIFNB1_LUC and 1,250 293T pEF1α-LUC in 100 μl of complete phenol-red free DMEM containing 4 μg/ml polybrene. Infection with lentivirus encoding shRNA were carried out immediately after cell seeding at a MOI of 10 (except when specified otherwise) and incubated for three days at 37°C in an atmosphere of 5% CO_2_. Cells were infected with 100 HAU/ml of SeV (Cantell Strain, Charles River Labs) for 16 hours before cell lysis and firefly luminescence reading in a 100 mM Tris acetate, 20 mM Mg acetate, 2 mM EGTA, 3.6 mM ATP, 1% Brij 58, 0.7% β-mercaptoethanol and 45 μg/ml luciferine pH 7.9 buffer. All infections were performed in an enclosed in a class II cabinet.

Assays used in western blot or qRT-PCR experiments where scaled up accordingly and carried out with the maternal HEK 293T or appropriate cell line.

### Influenza A/Gaussia luminescence assay

For influenza infection, 3x10^5^ HEK 293T pIFNB1_LUC cells were seeded in 6-well plates. The next day, cells were transfected with an influenza vRNA reporter plasmid [55] and infected with 0.1 ul of purified influenza virus (A/PR/8/34, from Charles River). Five days later, 20 ul of cell supernatant was used to quantify the Gaussia luciferase using a Gaussia Luciferase Assay HTS (Nanolight Technology). Cell lysates were used to quantify the IFNB1 induction according to the Firefly luminescence assay described above.

### HCV/Renilla luminescence assay

J6/JFH(p7-Rluc2a) virus production was conducted as previously described [56]. Briefly, HCV DNA template used for *in vitro* transcription was linearized using XbaI and subsequently transcribed using TranscriptAid T7 High Yield Transcription Kit according to manufacturer protocol (Life Technologies). The resulting HCV RNA was then electroporated into Huh7.5 and virus-containing culture medium was collected, filtered (0.45 μm) and kept at -80°C. For infection, 100μl of virus was added to 5,000 Huh7 cells that had been plated in 96-well white opaque plates one day before. Culture medium was replaced six hours later and Huh7 cells were transfected with pIFNB1-LUC (50 ng/well) the next day. Three days later Huh7 cells were washed twice with PBS, before Rluc and Fluc quantification using the Dual-Luciferase Reporter Assay System (Promega) according to the manufacturer protocol.

### Western immunoblot analysis

Cells were washed twice with ice-cold phosphate-buffered saline (PBS; Wisent), harvested and lysed in 10mM Tris-HCl, 100mM NaCl, 0.5% Triton X-100, pH7.6 with EDTA-free Protease Inhibitor Cocktail (Roche). Cell lysates were clarified by centrifugation at 13,000 g for 20 min at 4°C and subjected to sodium dodecyl sulfate-polyacrylamide gel (SDS-PAGE). Western blot analysis was performed using mouse anti-PHF5A (Abnova), anti-IRF3 (Santa Cruz), anti-TRAF3 (Santa Cruz), anti-RIG-I (Alexis Biochemicals), anti-ACTIN (Chemicon International), anti-TBK1 (Imgenex and Santa Cruz), anti-IKBKE (Santa Cruz), anti-TUBULIN (ICN), anti-GAPDH (RDI) and rabbit anti-SNRNP200 (Sigma-Aldrich), anti-SF3A1 (Santa Cruz), anti-RELA (Santa Cruz), anti-NHP2L1 (Abcam), anti-DDX3X (Bethyl), anti-DDX60 (Abcam), TRIF (Cell Signaling), anti-ISG56 (Novus Biologicals), anti-MDA5 (Alexis Biochemicals), anti-MAVS (Alexis Biochemicals), anti-IKBKE (eBioscience), STAT1 (ABCAM), STAT1 tyr701 (ABCAM), IFNAR1 (Santa Cruz) and anti-IRF3-P-ser386 (Abcam). HRP-conjugated secondary antibodies were from Bio-Rad. The chemiluminescence reaction was performed using the Western Lighting Chemiluminescence Reagent Plus (PerkinElmer).

### Co-immunoprecipitation

For co-immunoprecipitation, FLAG-tagged protein expressing cells were harvested and lysed as described above. Resulting cell extracts were adjusted to 1 mg/ml and subjected to IP as follows: preclearing of the lysates was done by incubating lysates with 40 μl of a 50:50 slurry of immunoglobulin G-Sepharose (GE Healthcare) prepared in the lysis buffer with IgG beads for 1 hour. Pre-cleared lysate were immunoprecipitated by adding 20 μl of M2 anti-FLAG affinity gel (Sigma-Aldrich) prepared in TBS buffer (50 mM Tris-HCl, 150 mM NaCl, pH 7.4) overnight as described by the manufacturer. Immunoprecipitates were washed five times in lysis buffer. For interaction analysis, elution was performed using 250 ng/μl purified FLAG peptide for 45 min at 4°C (Sigma-Aldrich). Eluates were analyzed by western immunoblotting.

### Microarray analysis

The microarray studies were performed with HEK 293T cells transduced with lentiviral-expressing shNT (control) or shSNRNP200 RNA targeting *SNRNP200* gene for three days following 16 hours infection with SeV (100 U/ml) or 16 hours of treatment with a mixture of IFN-α from human leukocytes (400 U/ml; Sigma-Aldrich). A total of 10 μg of RNA was reverse transcribed using oligo(dT) 16–18 primers and SuperScript II Reverse Transcriptase (Life Technologies) according to the manufacturer's instructions. Following purification using QIAquick PCR Purification kit (Qiagen), up to 1 μg of purified cDNA was mixed with 5'-Cy3 labeled random nonamers (Trilink Biotechnology) and heated at 95°C for 10 minutes and transferred on ice for 10 minutes. Samples were mixed with 1 mM dNTP and 2 μl of 3’-5’ exo-Klenow fragment (New England Biolabs) and incubated at 37°C for 2 hours. The labeling reaction was stopped using 50 μM EDTA and the DNA precipitated using 0.5 M NaCl and 1 volume isopropanol, washed with 80% ethanol and resuspended in water. Hybridizations were carried out using the Human GE 4x44K v2 Microarrays (Agilent Technologies) containing probes targeting 27,958 Entrez Gene RNAs. Arrays were scanned at 5 μm resolution using a GenePix4000B scanner (Molecular Devices). Data from scanned images were extracted using GenePix 6.1 (Axon) and processed and normalized using ArrayPipe (v2.0). Processed data was used as input for linear modeling using Bioconductor's limma package, which estimates the fold-change between predefined groups by fitting a linear model and using an empirical Bayes method to moderate standard errors of the estimated log-fold changes in expression values from each probe set. *P* values from the resulting comparison are adjusted for multiple testing according to the method of Benjamini and Hochberg. Subsequently, gene enrichment analysis are conducted using DAVID [57,58], STRING [59,60] and Gene networks were constructed using GENEMANIA [60].

### Biotin-RNA/Biotin-DNA pull-down

RNA pull-down assay was performed using Dynabeads M270 Streptavidin (Life Technologies). Dynabeads were incubated with biotin-labeled RNA (poly I:C (InvivoGen) and full-length Jc1 HCV) for 1 hours according to manufacturer’s protocol. Biotin-HCV RNA was obtained by subjecting linearized HCV DNA to T7 reverse transcription (TranscriptAid T7 High Yield, Life Technologies) and biotin-dUTP (Enzo Life Sciences). Saturated beads were added to whole 100 μg cell lysate and incubated, in a cold room, on a rotating wheel. Beads were washed three times and RNA-bound proteins were eluted after boiling in 0.1% SDS and analyzed by western blot. Poly (dA:dT) and Poly(dG:dC) were purchased from Sigma and labeled using Label IT Nucleic Acid Labeling Kit (Mirus Bio) and biotin-DNA pull-down assays were performed as described above.

### RNA extraction and qRT-PCR assays

Total cellular RNA was extracted with the RNeasy Mini kit (Qiagen). Reverse transcription was performed on 1 μg total cellular RNA using the High Capacity cDNA Reverse Transcription kit (Applied Biosystems). In order to amplify only the cDNA, primers were located in the splicing junction between two exons. PCR reactions were performed using 1.5 μl of cDNA samples (15 ng), 5 μl of the Fast TaqMan PCR Master Mix (Applied Biosystems), 10 pmol of each primer (IDT) and 5 pmol of the UPL probe (Roche) in a total volume of 10 μl. The ABI PRISM 7900HT Sequence Detection System (Applied Biosystems) was used to detect the amplification level and was programmed to an initial step of 3 minutes at 95°C, followed by 40 cycles of 5 seconds at 95°C, 30 seconds at 60°C and 1 second at 72°C. All reactions were run in duplicate on biological duplicate and the average values were used for quantification. ACTIN (β-actin) or GAPDH (Glyceraldehyde 3-phosphate dehydrogenase) and HPRT1 (hypoxanthine phosphoribosyltransferase 1) were used as endogenous controls. The relative quantification (RQ) of target genes was determined by using the ΔΔCt method with the Sequence Detection System (SDS) 2.2.2 software (Applied Biosystems).

### Virus plaque assays

Plaque assays were conducted in VERO cells and MDCK.2 cells (ATCC) using a method described elsewhere [61]. Briefly, supernatants were harvested from infected cells and used to inoculate in serial dilutions VERO (SeV) and MDCK.2 cells (FLUA) for 45 minutes and 1 hour, respectively. After infection, cells were wash with PBS and an overlay of 0,6% agarose was superimposed to 2X DMEM medium. At 72 hours post-infection, cells were colored with crystal violet, washed with PBS and colonies (lysed cells) were counted to compute viral titers.

### ELISA assays

ELISA assays were carried out with 50 μl of cell culture supernatants using the VeriKine Human Interferon Beta Elisa Kit (PBL Assay Science) according to the manufacturer’s protocol. Samples were run as technical duplicates on biological triplicates.

### Immunofluorescence analysis

HEK 293T were seeded in cover slip-containing 24-well plates and co-transfected with FLAG-SNRNP200 WT or S1087L mutant and MYC-TBK1 24 hours later. The following day, cells were infected or not with SeV for 16 hours before being washed twice with PBS, fixed with 4% paraformaldehyde-containing PBS during 20 minutes at room temperature and then permeabilized in 0.2% Triton X-100/PBS during 15 minutes. Blocking was made in PBS with 10% normal goat serum, 5% bovine serum albumin (BSA) and 0.02% sodium azide during 45 minutes at room temperature. Following three rapid washes, cells were labelled with rabbit anti-FLAG (Sigma-Aldrich) and mouse anti-MYC (Santa Cruz) primary antibodies diluted in 5% BSA/0.02% sodium azide/PBS for 2 hours. Slides were washed three times in PBS and then labeled with anti-rabbit or anti-mouse AlexaFluor 488, 594 or 647 secondary antibodies (Life Technologies) diluted in 5% BSA/0.02% sodium azide/PBS for 1 hour. Cells were extensively washed and incubated with Prolong Gold with DAPI (Life Technologies). Alternatively, nuclei were labeled with Syox Green (Life Technologies). Labelled cells were then examined by laser scanning microscopy using a TCS SP5 (Leica).

## Supporting Information

S1 Fig Screening of a subgroup of spliceosome members identified SNRNP200 as the only helicase required for the antiviral response of SeV infection. (A) HEK 293T pIFNB1-Luc cells are transduced with lentivirus-expressing shRNA targeting a subset of RNA helicases implicated in splicing for three days and stimulated with SeV for 16 hours. (B) HEK 293T are transduced with lentivirus-expressing shRNA targeting for 3 days or transfected for 48 hours with SNRNP200, SFRS1, SNRNP35, SF3A1, PHF5A and NHP2L1 expression plasmids. Protein KD and overexpression (OE) efficiencies of the various spliceosome proteins as well as IRF3, DDX58, IFIT1 and ACTIN protein levels are resolved by immunobloting of cell lysates and compared to shNT control cells. (C) HEK 293T are treated as indicated in (B) and infected with SeV for 16 hours.
(TIF)

S2 Fig SNRNP200 KD enhances viral replication and restricts antiviral response. (A) FLUA-Gaussia activity and IFNB1 promoter-driven luciferase activity of HEK 293T cells infected with FLUA for 24 hours and transduced with shNT or shSNRNP200 for three days. (B) HEK 293T cells are infected with FLUA for 24 and 48 hours and viral titers are determined by harvesting supernatants and subsequently infecting MDCK.2 cells using virus plaque assays. (C) HCV J6/JC1(2a)-Renilla luciferase activity and IFNB1 promoter-driven firefly luciferase activity of Huh7 cells transduced with shNT or shSNRNP200 for 4 days and infected with HCV for the three last days. P values <0.01 (**) or <0.001 (***) or <0.0001 (****) are indicated.
(TIF)

S3 Fig Silencing of SNRNP200 in A549 cells specifically inhibits activation of the RLR-dependent IFNB1 production and IFN-α signaling pathways, but does not affect activation of the canonical NF-κΒ pathway. (A) A549 cells treated with lentiviral-expressing shRNA targeting SNRNP200 or DDX58 at a multiplicity of infection (MOI) of 10 for three days. Relative IFN-β promoter activity are reported as percentage of the control shNT following infection with SeV for 8 hours or transfection of poly I:C, MAVS or IRF3(5D) for 16 hours. Inhibition profile of sh*SNRNP200* maps its site of action between MAVS and IRF3(5D) of the RLR signaling pathway. (B) Time course SeV infection (4, 8, 24 hours) in cells treated as indicated in (A). (C) qRT-PCR quantification of *IFIT1*, *IFIT2*, *DDX58*, *IFIH1*, *TNF*, *NFKBIA* and *TNFAIP3* mRNA fold induction in A549 cells transduced with lentiviral-expressing shNT (black bars) or shSNRNP200 (grey bars) for four days and treated with SeV or IFN-α for four hours. mRNA RQ were normalized versus *GAPDH* and *HPRT1* mRNA. *P* values <0.05 (*) are indicated.
(TIF)

S4 Fig SNRNP200 KD specifically inhibits activation of the RLR-dependent pathway, but does not affect activation of the canonical NF-κΒ pathway. (A) Relative NF-kB promoter-driven luciferase activity reported as percentage of the control shNT after transfection of HEK 293T cells with poly (I:C)/RIG-I, MAVS, TBK1 and p65 for 16 hours. (B) Relative ISG56 promoter-driven luciferase activity reported as percentage of the control shNT after SeV infection, transfection with TBK1, cGAS-STING and TRIF for 16 hours or IFN-α treatment.
(TIF)

S5 Fig SNRNP200 KD restricts SeV- and IFN-α-mediated induction of antiviral response and affects IRF3 expression (A) HEK 293T cells are transduced with shSNRNP200 for three days and then either unstimulated (NS), infected with SeV or stimulated with IFN-α for 16 hours. Cells are harvested and selected proteins including known members of the RLR signaling pathway (SNRNP200, IRF3, DDX58, IFIH1, IFIT1, IRF7, MAVS, TBK1, IKBKE, RELA, TRAF3, ACTIN, TUBULIN, GAPDH) are resolved by immunobloting of cell lysates and compared to shNT cells. (B) HEK 293T cells are treated as indicated in (A) and relative gene expression was measured by qRTPCR for *SNRNP200*, *DDX58*, *IRF3*, *IFIH1*, *IFIT1 and IFNB1* and compared to control shNT cells. Average mRNA RQ normalized versus *ACTIN* and *HPRT1* mRNA. P values <0.05 (*), <0.01(**) and <0.001 (***) are indicated.
(TIF)

S6 Fig Ectopic expression of IRF3 and DDX58 or both does not rescue antiviral response of SNRNP200 KD cells. (A) HEK 293T cells are transduced with shSNRNP200 for three days and transfected with DDX58 expression plasmid for the last 48 hours. Subsequently, cells are either untreated (NS), infected with SeV or stimulated with intracellular poly (I:C) for 16 hours. Cells are harvested and selected proteins (SNRNP200, DDX58, IRF3, IFIT1 and ACTIN) are resolved by immunobloting of cell lysates and compared to control shNT cells. (B) HEK 293T cells are transduced with shSNRNP200 for three days and transfected with DDX58 or IRF3 expression plasmids alone or in combination for the last 48 hours. Selected proteins are resolved as indicated in (A). (C) As a control experiment, unstimulated HEK 293T cells are transduced with shNT and transfected with SNRNP200 WT or S1087L variant expression plasmids for 48 hours. Cells are harvested and SNRNP200, DDX58, IFIT1, IRF3 and IRF3pS386 expression are resolved by immunobloting of cell lysates and compared to cells transfected with an empty expression plasmid (vector).
(TIF)

S7 Fig SNRNP200 KD does not induce *IRF3* mRNA alternative splicing. (A) Schematic representation of *IRF3* genomic organization and theoretical PCR products for the PCR exon spanning or junction strategies. Exons 1–7 are represented by black boxes and primers used for the PCR analysis are represented by arrows. (B) DNA electrophoresis of PCR products described in (A) after mRNA extraction and subjected to RT of HEK 293T cells transduced with shNT or shSNRNP200 for four days. Two independent experiments are presented. (C) qRT-PCR of IRF3 exon junctions described in (A) for exon 2–3 and exon 3–4 (left) and treated as indicated in (B). qRT-PCR of SNRNP200 and IRF3 following SNRNP200 KD (right). *P* values <0.05 (*) are indicated.
(TIF)

S8 Fig SNRNP200 silencing leads to an impaired induction of innate immunity genes. HEK 293T cells are transduced with shNT or shSNRNP200 for three days and either unstimulated (NS) infected with SeV or treated with IFN-α for 16 hours. Relative gene expression was measured by microarray and compared to control shNT cells. (A) Left—Volcano plot showing the effect of SNRNP200 silencing on gene expression level of untreated cells (SNRNP200_NS). Only genes > 1,5 log2 fold induction change are displayed. Right—Reactome Pathway Enrichment of up- or down-regulated genes upon SNRNP200 silencing. (B) Left—Venn diagram of the number of altered genes (> 1,5 log2 fold) of shSNRNP200 unstimulated cells (SNRNP200_NS) or infected with SeV (SNRNP200_SEV) and compared to control shNT cells infected with SeV (NT_SEV). Right—Volcano plot of the gene expression in shNT cells and shSNRNP200 following SeV infection. Table shows the gene ontology enrichment of the gene list used. (C) Left–Venn diagram of the number of altered genes (> 1,5 log2 fold) of shSNRNP200 unstimulated cells (SNRNP200_NS) or treated with IFN-α (SNRNP200_IFN-α) and compared to control shNT cells treated with IFN-α (NT_IFN-α). Right—Volcano plot of the gene expression in shNT cells and shSNRNP200 following IFN-α stimulation. Table shows the gene ontology enrichment of the gene list used.
(TIF)

S9 Fig The full-length protein sequence of SNRNP200 is required to rescue SeV-mediated induction of antiviral response in SNRNP200 KD cells. (A) HEK 293T pIFNB1-Luc cells are transduced with shSNRNP200 for three days and transfected with RNAi resistant expression plasmids for SNRNP200 WT, S1087L or C-terminal truncated mutants for the last 48 hours. Subsequently, cells are untreated (left panel) or infected with SeV for 16 hours (right panel). IFNB1 promoter-driven luciferase activities are measured and compared with control shNT cells. (B) HEK 293T cells are treated as indicated in (A). Cells are harvested and DDX58, IFIT1 and ACTIN proteins are resolved by immunobloting of cell lysates.
(TIF)

S10 Fig Ectopic expression of SNRNP200, but not Sec63-containing S1087L mutant, rescues SeV-mediated induction of *IFNB1* mRNA in SNRNP200 KD cells. (A) qRT-PCR quantification of *SNRNP200* and *IFNB1* mRNA levels of HEK 293T cells transduced with shSNRNP200 for three days and transfected with eYFP or RNAi resistant SNRNP200 WT or SNRNP200 S1087L expression plasmids for 48 hours and subjected to SeV infection for 16 hours.
(TIF)

S11 Fig SNRNP200 C502A variant elicits an IFNB1 response independently of viral infection. HEK 293T pIFNB1-Luc cells are transduced with shSNRNP200 for three days and transfected with RNAi resistant SNRNP200 WT or variants expression plasmids bearing the indicated mutation for 48 hours. IFNB1 promoter-driven luciferase activities are measured and compared with control shNT cells.
(TIF)

S12 Fig Constitutive interaction of TBK1 and SNRNP200 endogenous proteins in A549 cells. (A) A549 cells are untreated or infected with SeV for 16 hours. Cell lysates are subjected to immunoprecipitation using anti-TBK1 or control IgG antibodies followed by incubation with protein G sepharose beads. TBK1 and SNRNP200 are resolved by immunoblotting of immune complexes (up) and cell lysates (down). Results are compared to untreated cells. TBK1 protein is indicated with an asterix.
(TIF)

S13 Fig Relocalization of SNRNP200 into cytoplasmic speckles and co-staining with TBK1 is dependent on SeV infection in Hela cells. (A) Hela cells are stained with anti-TBK1 and anti-SNRNP200 antibodies and analyzed by confocal microscopy. Nuclei are stained with Sytox Green. A merge for both protein is shown (Merge G/R) and at higher magnification (down panel). SNRNP200 staining is green and TBK1 is red. White arrows indicate TBK1 stained as red dots. Imaging was done using a 63x/1.40 Oil DIC objective. (B) Hela cells are infected with SeV for 16 hours and analyzed as indicated in (B). White arrows indicate SNRNP200-TBK1 complex stained as yellow dots. Imaging was done using a 63x/1.40 Oil DIC objective.
(TIF)

S14 Fig SNRNP200 protein accumulation in HEK 293T following SeV infection or IFN-α treatment does not result from an increase in mRNA levels. (A) Immunoblot analysis of HEK 293T cells infected with SeV or treated with IFN-α for the indicated times. (B) qRT-PCR quantification of *SNRNP200* mRNA levels of HEK 293T cells treated as indicated in (A).
(TIF)

S15 Fig PBMCs from RP33 patients bearing monoallelic point mutation in SNRNP200 show hindered IFN-α2 secretion. (A) PBMCs of RP33 patients (RP33) or healthy donors (HD) are infected with SeV for 16 hours. Supernatants are then harvested and cytokine levels are measured by multiplex-ELISA. In total 42 cytokines are analyzed and representative results for IFN-α2, RANTES, IL6, CXCL10 and IL1B are shown.
(TIF)

S16 Fig DDX58 binds biotinylated poly (I:C) *in vitro*. (A) HEK 293T cells are transfected with FLAG-DDX58 expression plasmid for 48 hours and infected with SeV for 16 hours. RNA pull-down assays are performed on cell lysates using biotinylated poly (I:C). Cell lysates and bead-bound complexes are analyzed by Western blotting and compared to uninfected control cells.
(TIF)

S1 Table Description of the RP33 patients (Age, Sex, Ethnicity and Retinitis Pigmentosa associated polymorphism) who volunteered PBMCs used in the experiments presented in Fig 9. Mean ages of patients and healthy donors were matched (43.3 vs. 43.0).
(TIF)

S2 Table Excel workbook describing the transcriptional profiling analysis of SNRNP200 KD cells as presented in Fig 3. The workbook contains the raw data (expression fold change, gene list, Genemania network and function enrichment analysis) of the common gene subset from Fig 3B, of the SeV-specific gene subset from Fig 3C and of the IFNα-specific gene subset from Fig 3D.
(XLSX)
