# Correction: Spliceosome SNRNP200 Promotes Viral RNA Sensing and IRF3 Activation of Antiviral Response

**DOI:** 10.1371/journal.ppat.1006174

**Published:** 2017-01-24

**Authors:** Nicolas Tremblay, Martin Baril, Laurent Chatel-Chaix, Salwa Es-Saad, Alex Young Park, Robert K. Koenekoop, Daniel Lamarre

The authors would like to correct Figures 1, 6, and 8, as changes were made to these figures in preparation for publication that were not indicated in the original figures and figure legends. In addition, some of those changes resulted in errors in the presentation of the final figures.

In Figure 1C cropping was done during image preparation, and a vertical line should have been inserted in the western blot presented to indicate the cropping. Figure 1C was assembled from a larger experiment, which included a 72-hour time point infection, as shown in the un-cropped blot in S1 Fig here. The corrected Fig 1C has been modified to show the above mentioned modification. The raw blots for Fig 1C are also presented here in S1 Fig.

**Fig 1:**
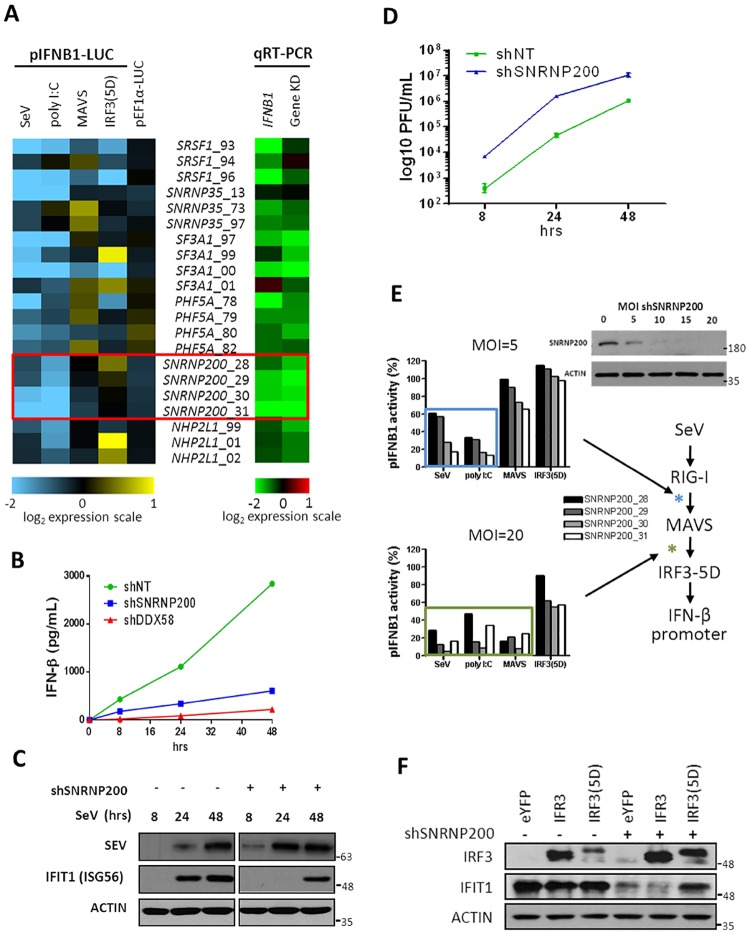
SNRNP200 spliceosome protein is required for virus-induced IFNB1 production to control viral replication. (A) HEK 293T cells stably expressing an IFNB1promoter-driven luciferase gene (HEK 293T pIFNB1-Luc) are transduced with different lentiviral-expressing shRNA targeting SNRNP200, SFRS1, SNRNP35, SF3A1, PHF5A and NHP2L1 genes. Left panel—Heat map (log2 scale) indicating the modulation of IFNB1 promoter activity following silencing of spliceosome genes and infection with SeV or transfection of poly I:C, MAVS or IRF3(5D) expression plasmids for 16 hours. Right panel—qRT-PCR validation data of the endogenous IFNB1 mRNA levels and target gene KD efficiency of cells transduced with shRNA. (B) HEK 293T are transduced with lentiviral-expressing shRNA control (shNT) or targeting SNRNP200 (shSNRNP200) or DDX58 (shDDX58) for three days and infected cells with SeV for 8, 24 or 48 hours. Supernatants are harvested and IFN-β secretion levels are measured by ELISA. (C) Immunoblot analysis of HEK 293T cells infected with SeV for 8, 24 or 48 hours following treatment with shNT or shSNRNP200 for three days. SeV, IFIT1 and actin proteins are resolved by immunobloting at the indicated time. For clarity, the 72-hour time point infection was removed from the figure. Raw blots are presented in S1 Fig. (D) Infectivity titers of SeV particles produced as indicated in (C) are determined by harvesting supernatants at the indicated time and infecting VERO cells in virus plaque assays. (E) HEK 293T pIFNB1-Luc cells are transduced with four different shSNRNP200 at a multiplicity of infection (MOI) of 5 and 20 for three days. Relative IFNB1 promoter activity are reported as percentage of the control shRNA NT after infection with SeV or transfection of poly I:C, MAVS or IRF3(5D) expression plasmids for 16 hours (left). Simplified schematic of RLR signaling pathway leading to IFN-β promoter induction (right). Deduced points of action of SNRNP200 are marked with asterisks (blue and green for MOI = 5 and 20, respectively). Knockdown efficiencies at the various MOI are determined by immunobloting analysis of SNRNP200 protein levels. (F) Immunoblot analysis of HEK 293T cells transduced with shNT or shSNRNP200 for three days and subjected to SeV infection for 16 hours. Plasmids encoding eYFP, IRF3 and IRF3(5D) are transfected for 48 hours. Following cell harvesting, IRF3 and IFIT1 protein levels are resolved by immunobloting analysis of cell lysates.

In Figures 6A, 6B, and 6C cropping was done during image preparation and vertical lines should have been inserted in the western blot presented to indicate the cropping. Figures 6A, 6B, and 6C were generated from a larger experiment as shown in the un-cropped blot in S2 Fig. For the corrected Fig 6A, the authors performed a new experiment as described in the original manuscript, which is now presented here. Vertical lines have been inserted to the corrected Fig 6B to show where cropping occurred. The corrected Fig 6C was reorganized to show where the cropping occurred. The raw blots for Fig 6A, 6B and 6C are also presented here in S2 Fig.

**Fig 6:**
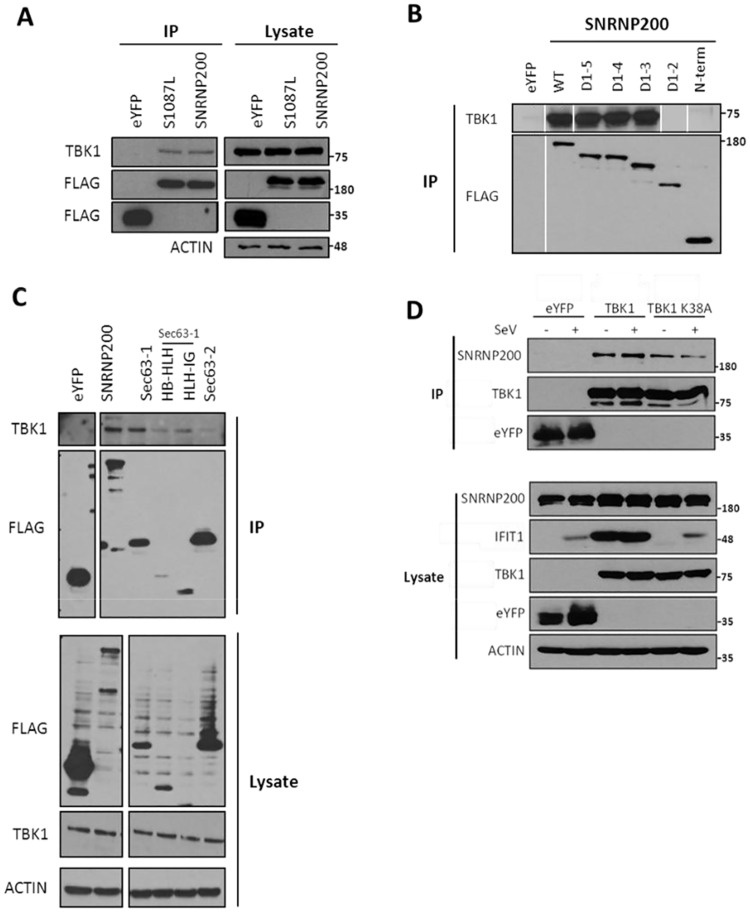
SNRNP200 Sec63-1 domain interacts with TBK1. (A) HEK 293T cells are transfected with FLAG-eYFP (control), FLAG-SNRNP200 or FLAG-SNRNP200 S1087L mutant expressing plasmids for 48 hours. Cell lysates are prepared following 16 hours of SeV infection and subjected to immunoprecipitation with anti-FLAG antibodies. Cell lysates and immune complexes are resolved by immunobloting analysis using anti-FLAG and anti-TBK1 antibodies. Raw blots are presented in S2 Fig. (B) Immunoprecipitation of FLAG-SNRNP200 C-terminal deletion mutants are performed and analyzed as indicated in (A). Vertical lines indicate that cropping was done on the blots from a larger experiment to remove unnecessary lanes and enhance clarity. Raw blots are presented in S2 Fig. (C) Immunoprecipitation of FLAG-SNRNP200 Sec63-1, HB-HLH or HLH-IG subdomains of Sec63-1 and Sec63-2 are performed and analyzed as indicated in (A). Cropping was done on the blots from a larger experiment to remove unnecessary lanes and enhance clarity. Raw blots are presented in S2 Fig. (D) Reciprocal immunoprecipitation of FLAG-eYFP (control), FLAG-TBK1 or FLAG-TBK1 K38A mutant following ectopic expression of SNRNP200 are performed as indicated in (A) and analyzed as indicated in (A). Cell lysates and immune complexes are resolved by immunobloting analysis using anti-FLAG and anti-SNRNP200 antibodies.

In Figures 8A and 8B cropping was done during image preparation, which resulted in the duplication of an actin panel in Figure 8B. Figures 8A and 8B were generated from a larger experiment as shown in the un-cropped blot in S3 Fig. For the corrected Fig 8A and 8B, the authors have provided a new Fig 8 that includes an A/B panel, which is made from the complete unspliced data. The raw blots for Fig 8 are presented here in S3 Fig.

**Fig 8:**
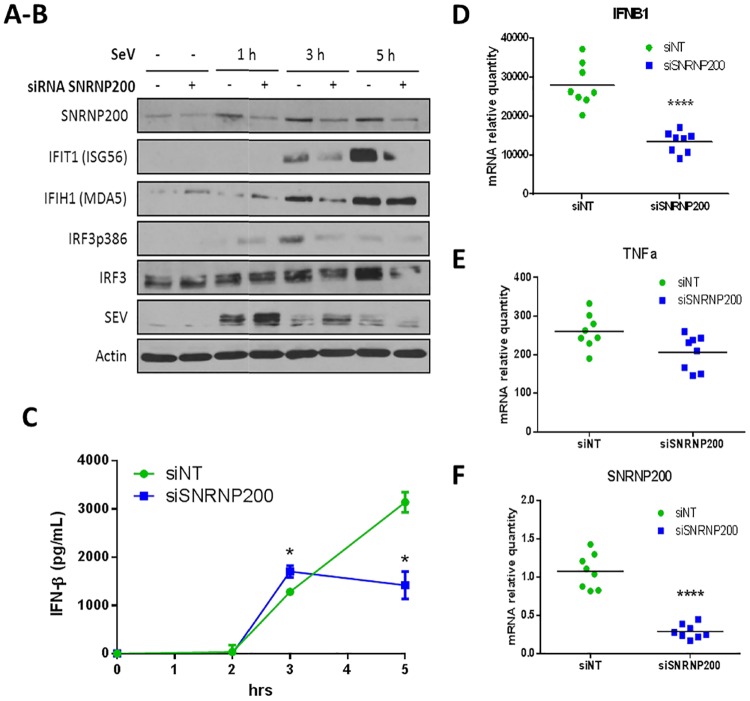
SNRNP200 KD restricts SeV-mediated antiviral response of human MDM. (A-B) MDM are transfected with a pool of siRNA targeting SNRNP200 for 48 hours and infected with SeV. For 1, 3 and 5 hours, cells are harvested and selected proteins (SNRNP200, IFIT1, IFIH1, IRF3, IRF3-p386, SeV and actin) are resolved by immunobloting of cells lysates and compared to control cells treated with scrambled siRNA. Raw blots are presented in S3 Fig. (C) MDM are transfected with a pool of siRNA targeting SNRNP200 for 48 hours and infected with SeV for 1, 3 or 5 hours. Supernatants are harvested and IFN-β secretion levels are measured by ELISA and compared to control cells treated with scrambled siRNA (siNT). (D-F) MDM are transfected with a pool of siRNA targeting SNRNP200 (siSNRNP200) or scrambled siRNA (siNT) and infected with SeV for 1 hour. Cells are harvested and relative gene expression of IFNB1 (D), TNFα (E) and SNRNP200 (F) are measured by qRT-PCR and compared with scrambled control cells. mRNA RQ are normalized versus ACTIN and HPRT1 mRNA. P values <0.0001 (****) are indicated. Data are pooled results from two experiments of two biological replicates.

The authors confirm that these changes do not alter their findings. The authors have provided raw, uncropped blots as Supporting Information.

## Supporting Information

S1 Fig Uncropped blots of Fig 1C. Immunoblot analysis of HEK 293T cells infected with SeV for 8, 24, 48 or 72 hours following treatment with shNT or shSNRNP200 for three days. SeV and IFIT1 proteins are resolved by immunobloting at the indicated time. For clarity, the lane for the 72-hour time point infection was removed of Fig 1C. Red boxes indicate the cropped area used in Fig 1C. The immunobloting of actin is not available as the raw data could not be retrieved.
(TIF)

S2 Fig Uncropped blots of Figs 6A, 6B and 6C. (A) HEK 293T cells are transfected with FLAG-eYFP (control), FLAG-SNRNP200 or FLAG-SNRNP200 S1087L mutant expressing plasmids for 48 hours. Cell lysates are prepared following 16 hours of SeV infection and subjected to immunoprecipitation with anti-FLAG antibodies. Cell lysates and immune complexes are resolved by immunobloting analysis using anti-FLAG and anti-TBK1 antibodies. Red boxes indicate the cropped area used in Fig 6A. (B) Immunoprecipitation of FLAG-SNRNP200 C-terminal deletion mutants. HEK 293T cells are transfected with FLAG-eYFP (control), FLAG-SNRNP200 and FLAG-deletion mutants expressing plasmids for 48 hours. Cell lysates are prepared following 16 hours of SeV infection and subjected to immunoprecipitation with anti-FLAG antibodies. Immune complexes are resolved by immunobloting analysis using anti-FLAG and anti-TBK1 antibodies. Left. Cropping was done on the blots to remove lanes of the larger experiment. Right. Cropping was done to enhance clarity and match the presentation of the overall figure. Red boxes indicate the cropped area used in Fig 6B. (C) Immunoprecipitation of FLAG-SNRNP200 Sec63-1, HB-HLH or HLH-IG subdomains of Sec63-1 and Sec63-2 are performed and analyzed as indicated in (A). Cropping was done on the blots from a larger experiment to remove unnecessary lanes and enhance clarity. Red boxes indicate the cropped area used in Fig 6C.
(TIF)

S3 Fig Uncropped blots of Fig 8A-B. (A-B) MDM are transfected with a pool of siRNA targeting SNRNP200 for 48 hours and infected with SeV. At 1, 3 and 5 hours post-infection, cells are harvested and selected proteins (SNRNP200, IFIT1, IFIH1, IRF3, IRF3-p386, SeV and actin) are resolved by immunobloting of cells lysates and compared to control cells treated with scrambled siRNA. Red boxes indicate the cropped area used in Fig 8A-B.
(TIF)
