# Supplementary figures and images for: Spliceosome SNRNP200 Promotes Viral RNA Sensing and IRF3 Activation of Antiviral Response
Source: PLoS Pathog. 2016 Jul 25;12(7):e1005772. doi: 10.1371/journal.ppat.1005772 (PMC4959778; doi:10.1371/journal.ppat.1005772)

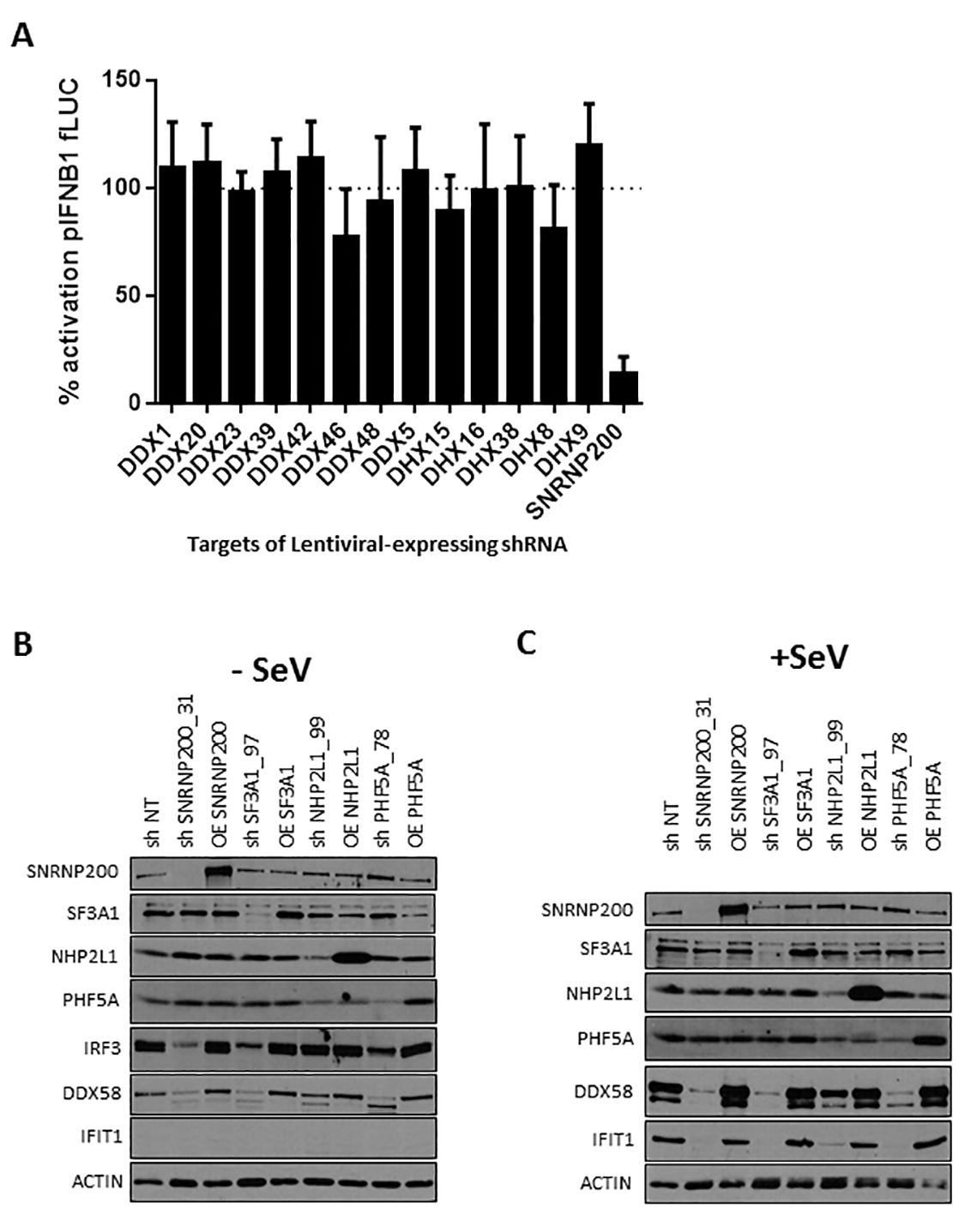

Supplement: S1 Fig — (A) HEK 293T pIFNB1-Luc cells are transduced with lentivirus-expressing shRNA targeting a subset of RNA helicases implicated in splicing for three days and stimulated with SeV for 16 hours. (B) HEK 293T are transduced with lentivirus-expressing shRNA targeting for 3 days or transfected for 48 hours with SNRNP200, SFRS1, SNRNP35, SF3A1, PHF5A and NHP2L1 expression plasmids. Protein KD and overexpression (OE) efficiencies of the various spliceosome proteins as well as IRF3, DDX58, IFIT1 and ACTIN protein levels are resolved by immunobloting of cell lysates and compared to shNT control cells. (C) HEK 293T are treated as indicated in (B) and infected with SeV for 16 hours. (TIF) [file ppat.1005772.s001.tif]

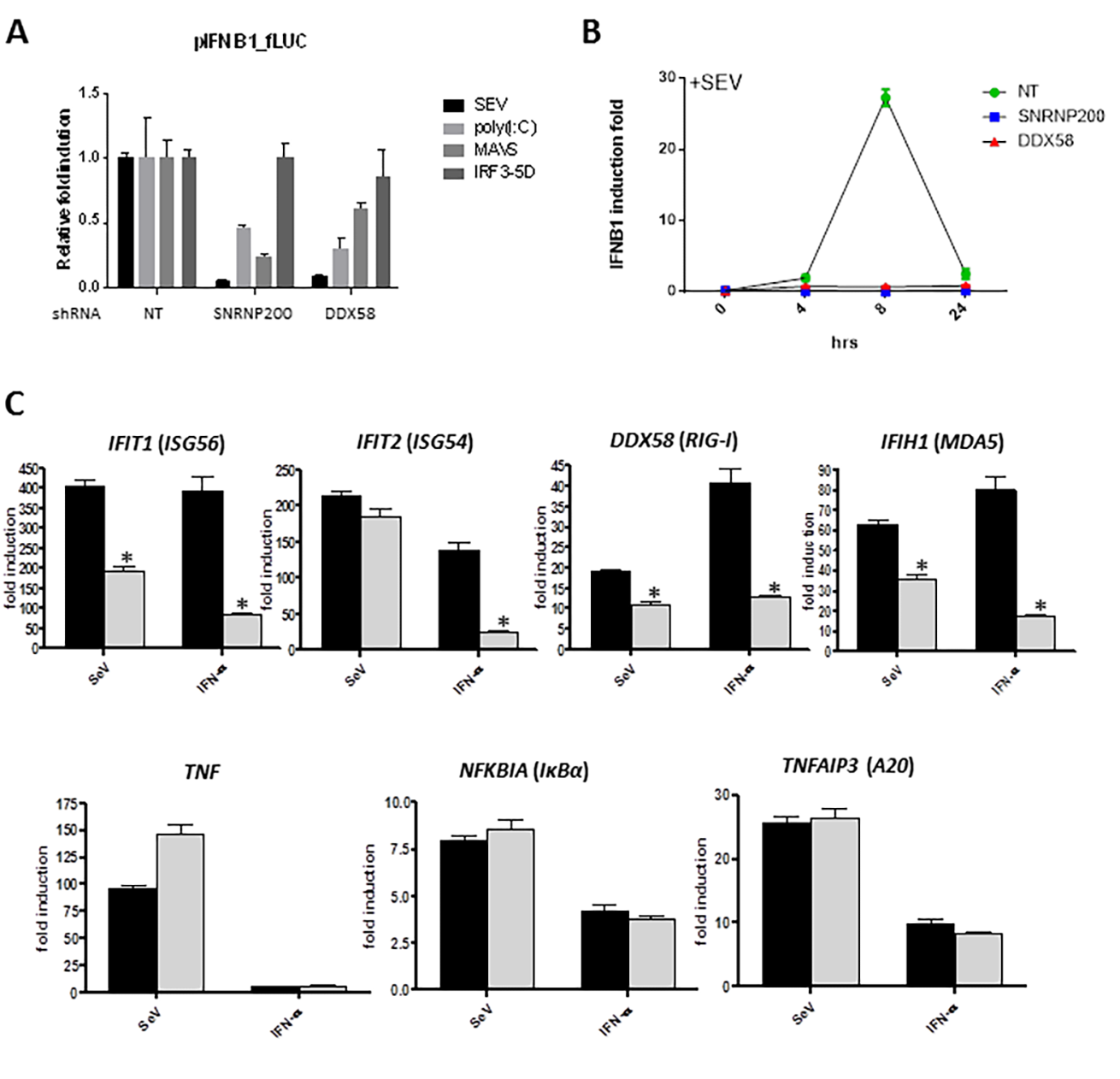

Supplement: S3 Fig — (A) A549 cells treated with lentiviral-expressing shRNA targeting SNRNP200 or DDX58 at a multiplicity of infection (MOI) of 10 for three days. Relative IFN-β promoter activity are reported as percentage of the control shNT following infection with SeV for 8 hours or transfection of poly I:C, MAVS or IRF3(5D) for 16 hours. Inhibition profile of shSNRNP200 maps its site of action between MAVS and IRF3(5D) of the RLR signaling pathway. (B) Time course SeV infection (4, 8, 24 hours) in cells treated as indicated in (A). (C) qRT-PCR quantification of IFIT1, IFIT2, DDX58, IFIH1, TNF, NFKBIA and TNFAIP3 mRNA fold induction in A549 cells transduced with lentiviral-expressing shNT (black bars) or shSNRNP200 (grey bars) for four days and treated with SeV or IFN-α for four hours. mRNA RQ were normalized versus GAPDH and HPRT1 mRNA. P values <0.05 (*) are indicated. (TIF) [file ppat.1005772.s003.tif]

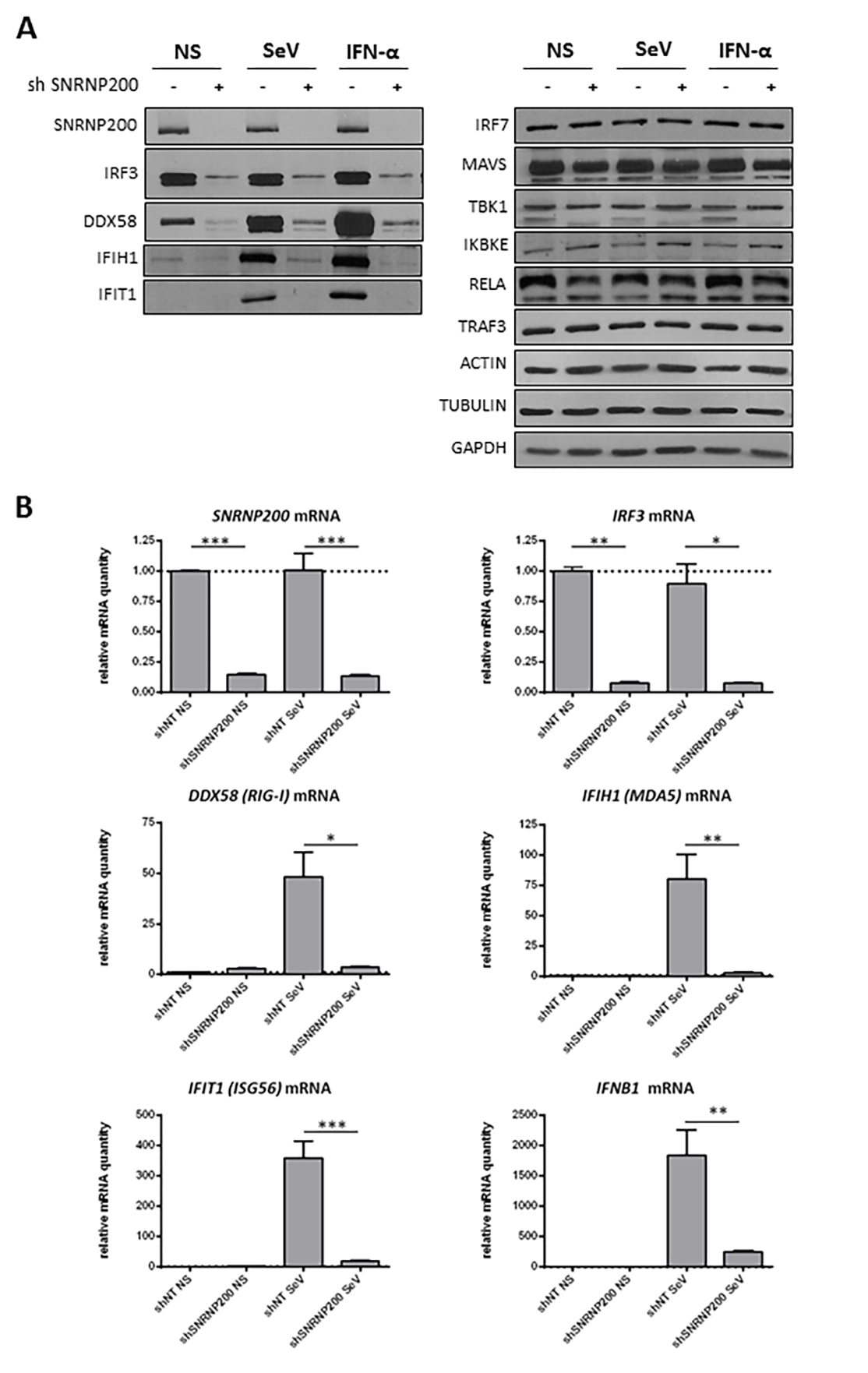

Supplement: S5 Fig — SNRNP200 KD restricts SeV- and IFN-α-mediated induction of antiviral response and affects IRF3 expression (A) HEK 293T cells are transduced with shSNRNP200 for three days and then either unstimulated (NS), infected with SeV or stimulated with IFN-α for 16 hours. Cells are harvested and selected proteins including known members of the RLR signaling pathway (SNRNP200, IRF3, DDX58, IFIH1, IFIT1, IRF7, MAVS, TBK1, IKBKE, RELA, TRAF3, ACTIN, TUBULIN, GAPDH) are resolved by immunobloting of cell lysates and compared to shNT cells. (B) HEK 293T cells are treated as indicated in (A) and relative gene expression was measured by qRTPCR for SNRNP200, DDX58, IRF3, IFIH1, IFIT1 and IFNB1 and compared to control shNT cells. Average mRNA RQ normalized versus ACTIN and HPRT1 mRNA. P values <0.05 (*), <0.01(**) and <0.001 (***) are indicated. (TIF) [file ppat.1005772.s005.tif]

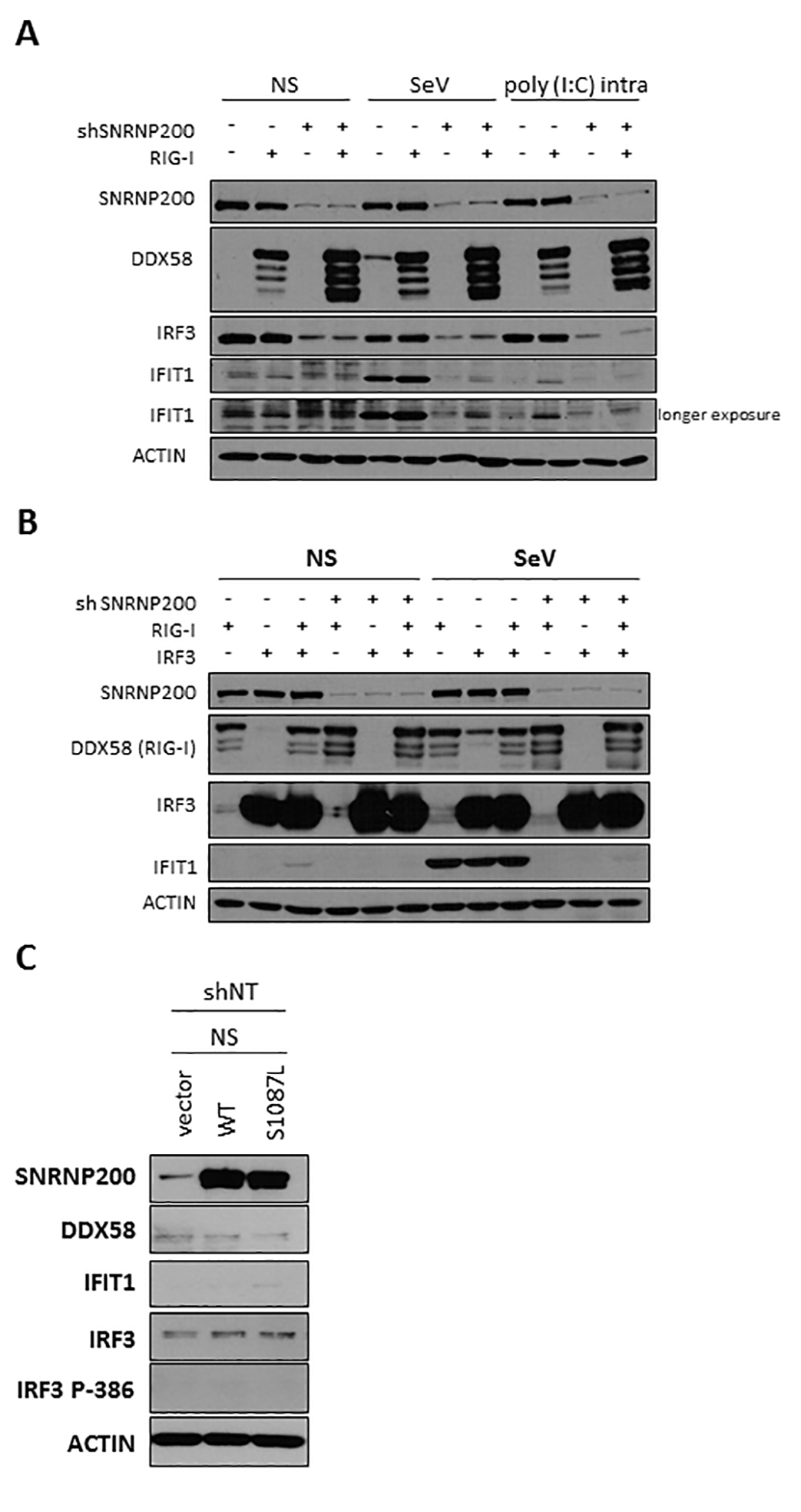

Supplement: S6 Fig — (A) HEK 293T cells are transduced with shSNRNP200 for three days and transfected with DDX58 expression plasmid for the last 48 hours. Subsequently, cells are either untreated (NS), infected with SeV or stimulated with intracellular poly (I:C) for 16 hours. Cells are harvested and selected proteins (SNRNP200, DDX58, IRF3, IFIT1 and ACTIN) are resolved by immunobloting of cell lysates and compared to control shNT cells. (B) HEK 293T cells are transduced with shSNRNP200 for three days and transfected with DDX58 or IRF3 expression plasmids alone or in combination for the last 48 hours. Selected proteins are resolved as indicated in (A). (C) As a control experiment, unstimulated HEK 293T cells are transduced with shNT and transfected with SNRNP200 WT or S1087L variant expression plasmids for 48 hours. Cells are harvested and SNRNP200, DDX58, IFIT1, IRF3 and IRF3pS386 expression are resolved by immunobloting of cell lysates and compared to cells transfected with an empty expression plasmid (vector). (TIF) [file ppat.1005772.s006.tif]

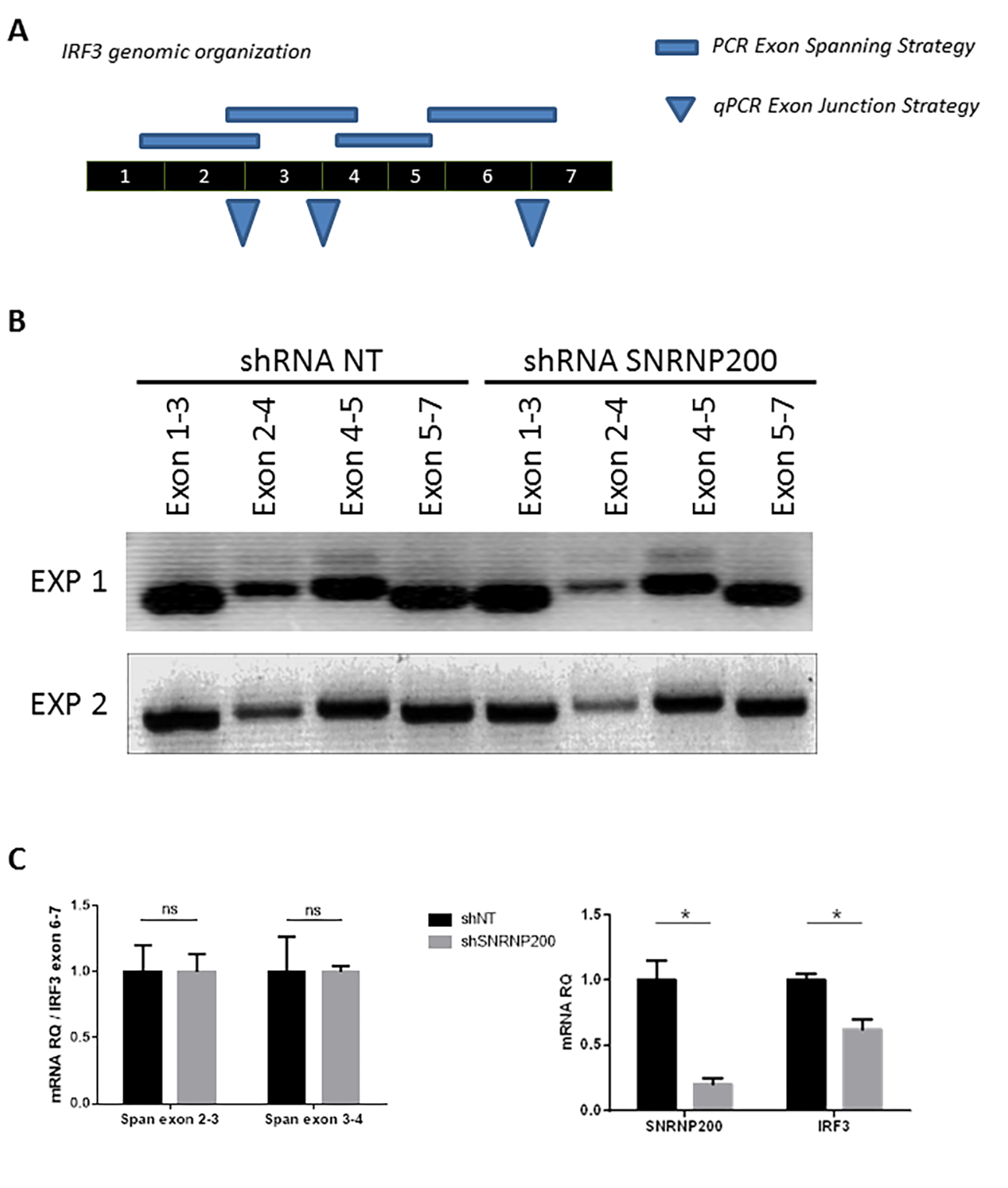

Supplement: S7 Fig — (A) Schematic representation of IRF3 genomic organization and theoretical PCR products for the PCR exon spanning or junction strategies. Exons 1–7 are represented by black boxes and primers used for the PCR analysis are represented by arrows. (B) DNA electrophoresis of PCR products described in (A) after mRNA extraction and subjected to RT of HEK 293T cells transduced with shNT or shSNRNP200 for four days. Two independent experiments are presented. (C) qRT-PCR of IRF3 exon junctions described in (A) for exon 2–3 and exon 3–4 (left) and treated as indicated in (B). qRT-PCR of SNRNP200 and IRF3 following SNRNP200 KD (right). P values <0.05 (*) are indicated. (TIF) [file ppat.1005772.s007.tif]

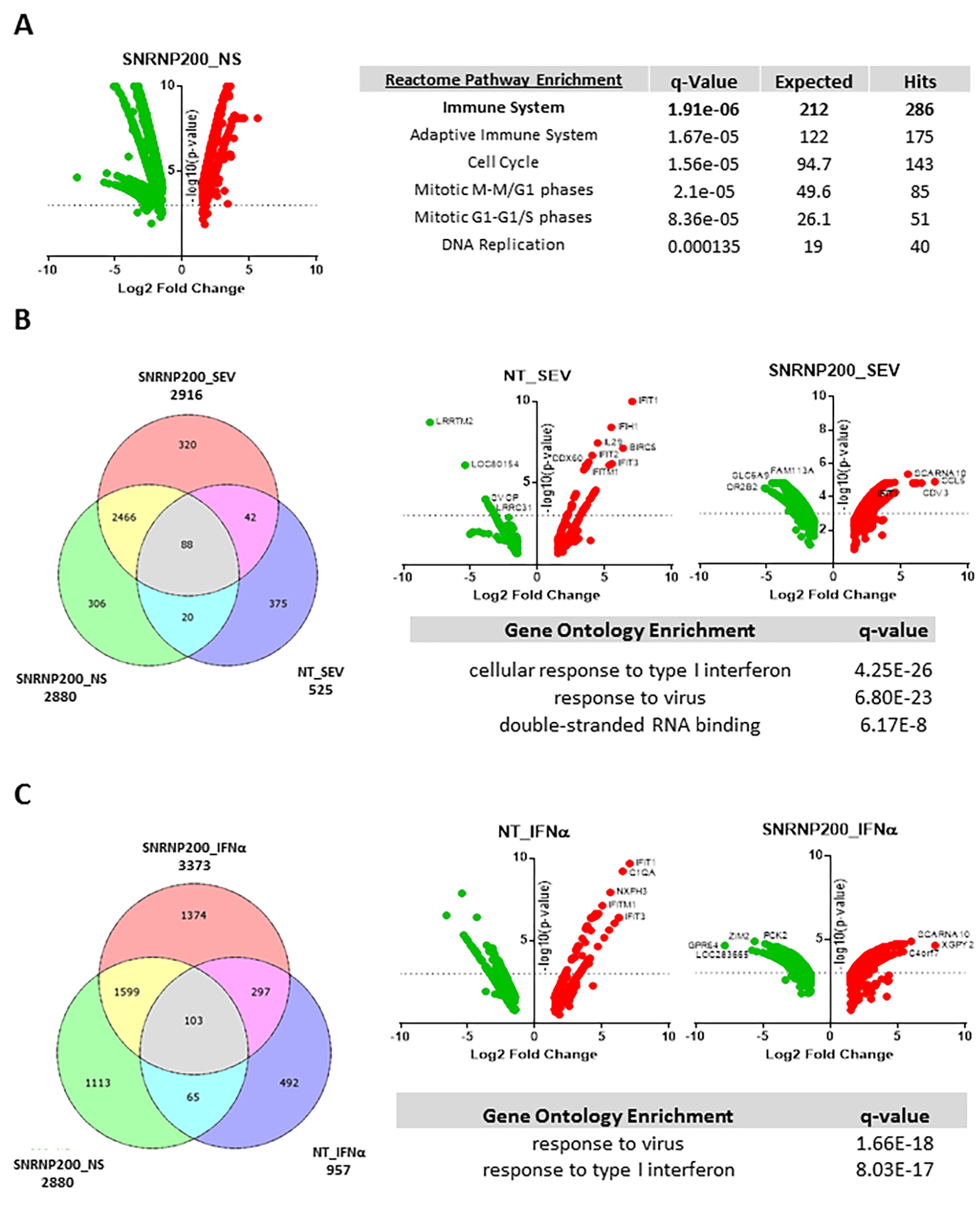

Supplement: S8 Fig — HEK 293T cells are transduced with shNT or shSNRNP200 for three days and either unstimulated (NS) infected with SeV or treated with IFN-α for 16 hours. Relative gene expression was measured by microarray and compared to control shNT cells. (A) Left—Volcano plot showing the effect of SNRNP200 silencing on gene expression level of untreated cells (SNRNP200_NS). Only genes > 1,5 log2 fold induction change are displayed. Right—Reactome Pathway Enrichment of up- or down-regulated genes upon SNRNP200 silencing. (B) Left—Venn diagram of the number of altered genes (> 1,5 log2 fold) of shSNRNP200 unstimulated cells (SNRNP200_NS) or infected with SeV (SNRNP200_SEV) and compared to control shNT cells infected with SeV (NT_SEV). Right—Volcano plot of the gene expression in shNT cells and shSNRNP200 following SeV infection. Table shows the gene ontology enrichment of the gene list used. (C) Left–Venn diagram of the number of altered genes (> 1,5 log2 fold) of shSNRNP200 unstimulated cells (SNRNP200_NS) or treated with IFN-α (SNRNP200_IFN-α) and compared to control shNT cells treated with IFN-α (NT_IFN-α). Right—Volcano plot of the gene expression in shNT cells and shSNRNP200 following IFN-α stimulation. Table shows the gene ontology enrichment of the gene list used. (TIF) [file ppat.1005772.s008.tif]

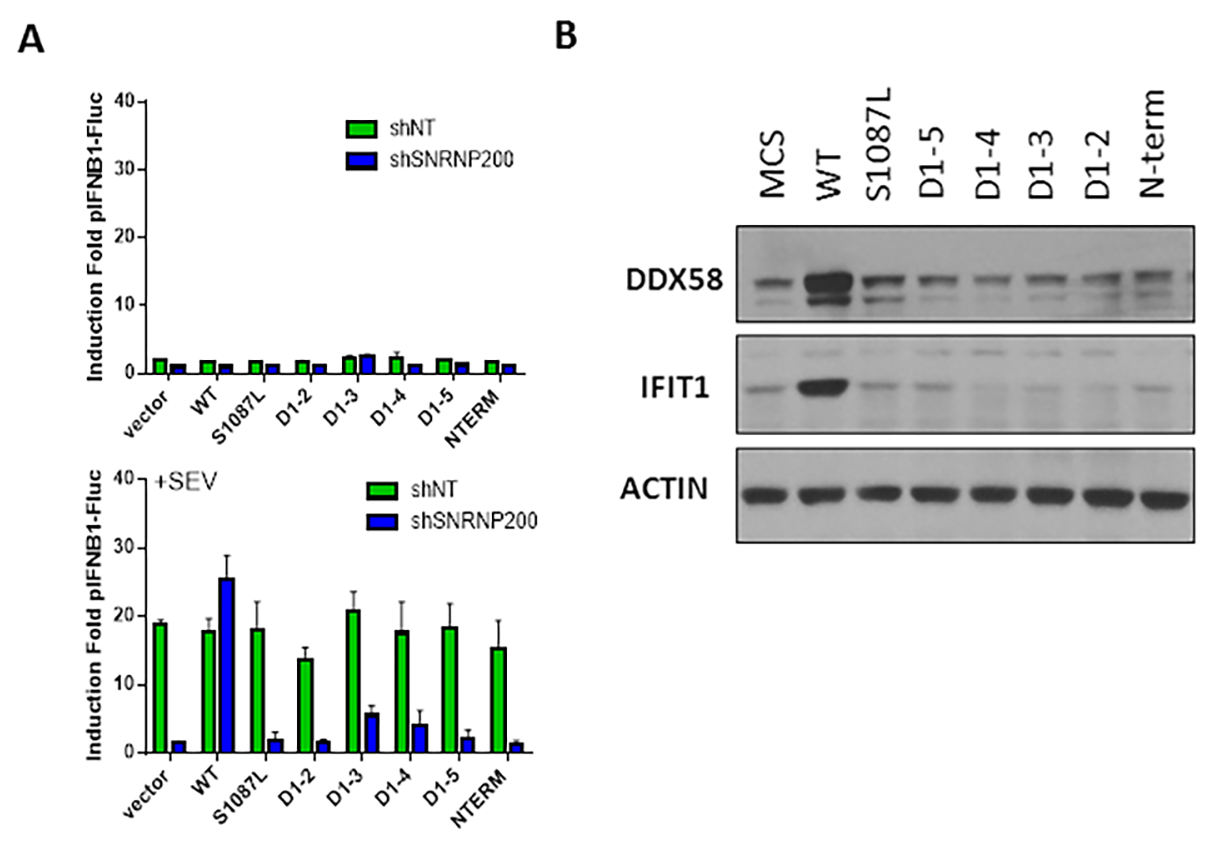

Supplement: S9 Fig — (A) HEK 293T pIFNB1-Luc cells are transduced with shSNRNP200 for three days and transfected with RNAi resistant expression plasmids for SNRNP200 WT, S1087L or C-terminal truncated mutants for the last 48 hours. Subsequently, cells are untreated (left panel) or infected with SeV for 16 hours (right panel). IFNB1 promoter-driven luciferase activities are measured and compared with control shNT cells. (B) HEK 293T cells are treated as indicated in (A). Cells are harvested and DDX58, IFIT1 and ACTIN proteins are resolved by immunobloting of cell lysates. (TIF) [file ppat.1005772.s009.tif]

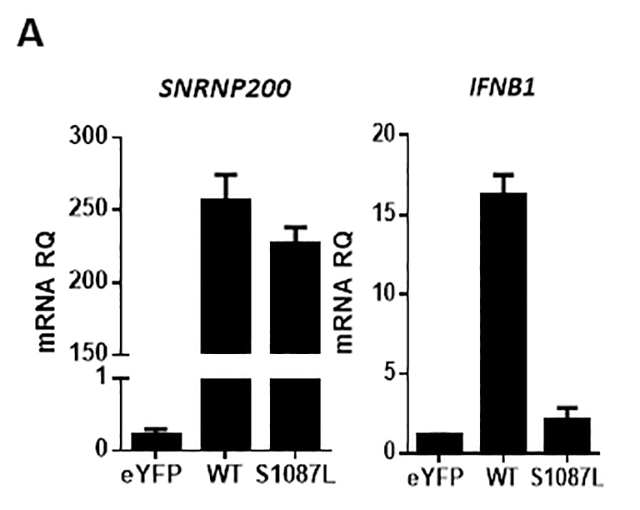

Supplement: S10 Fig — (A) qRT-PCR quantification of SNRNP200 and IFNB1 mRNA levels of HEK 293T cells transduced with shSNRNP200 for three days and transfected with eYFP or RNAi resistant SNRNP200 WT or SNRNP200 S1087L expression plasmids for 48 hours and subjected to SeV infection for 16 hours. (TIF) [file ppat.1005772.s010.tif]

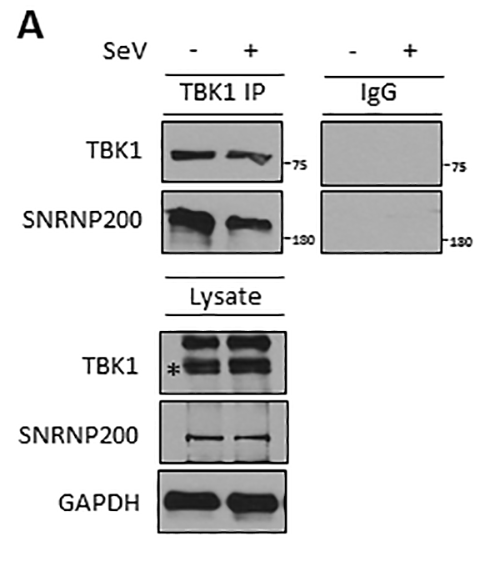

Supplement: S12 Fig — (A) A549 cells are untreated or infected with SeV for 16 hours. Cell lysates are subjected to immunoprecipitation using anti-TBK1 or control IgG antibodies followed by incubation with protein G sepharose beads. TBK1 and SNRNP200 are resolved by immunoblotting of immune complexes (up) and cell lysates (down). Results are compared to untreated cells. TBK1 protein is indicated with an asterix. (TIF) [file ppat.1005772.s012.tif]

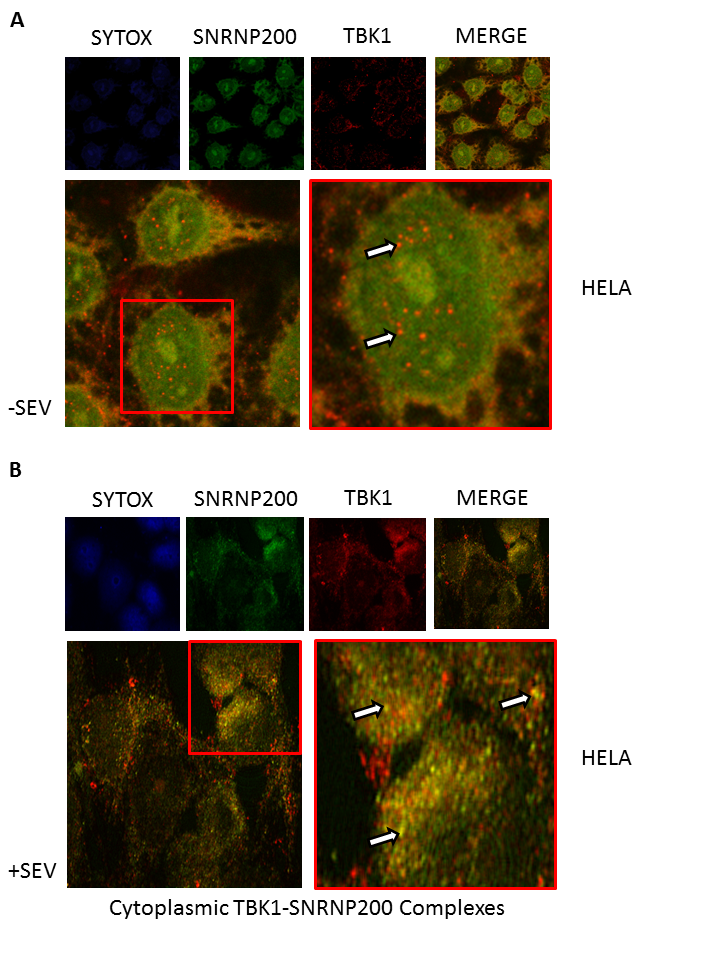

Supplement: S13 Fig — (A) Hela cells are stained with anti-TBK1 and anti-SNRNP200 antibodies and analyzed by confocal microscopy. Nuclei are stained with Sytox Green. A merge for both protein is shown (Merge G/R) and at higher magnification (down panel). SNRNP200 staining is green and TBK1 is red. White arrows indicate TBK1 stained as red dots. Imaging was done using a 63x/1.40 Oil DIC objective. (B) Hela cells are infected with SeV for 16 hours and analyzed as indicated in (B). White arrows indicate SNRNP200-TBK1 complex stained as yellow dots. Imaging was done using a 63x/1.40 Oil DIC objective. (TIF) [file ppat.1005772.s013.tif]

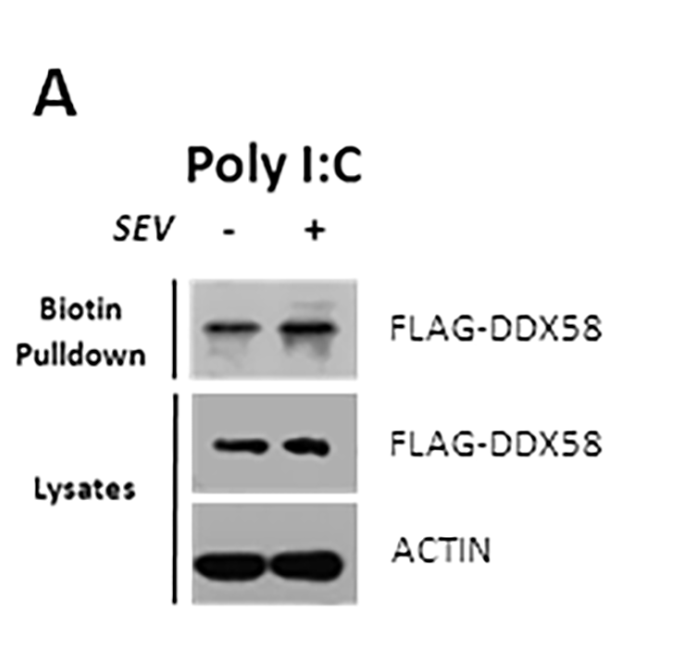

Supplement: S16 Fig — (A) HEK 293T cells are transfected with FLAG-DDX58 expression plasmid for 48 hours and infected with SeV for 16 hours. RNA pull-down assays are performed on cell lysates using biotinylated poly (I:C). Cell lysates and bead-bound complexes are analyzed by Western blotting and compared to uninfected control cells. (TIF) [file ppat.1005772.s016.tif]

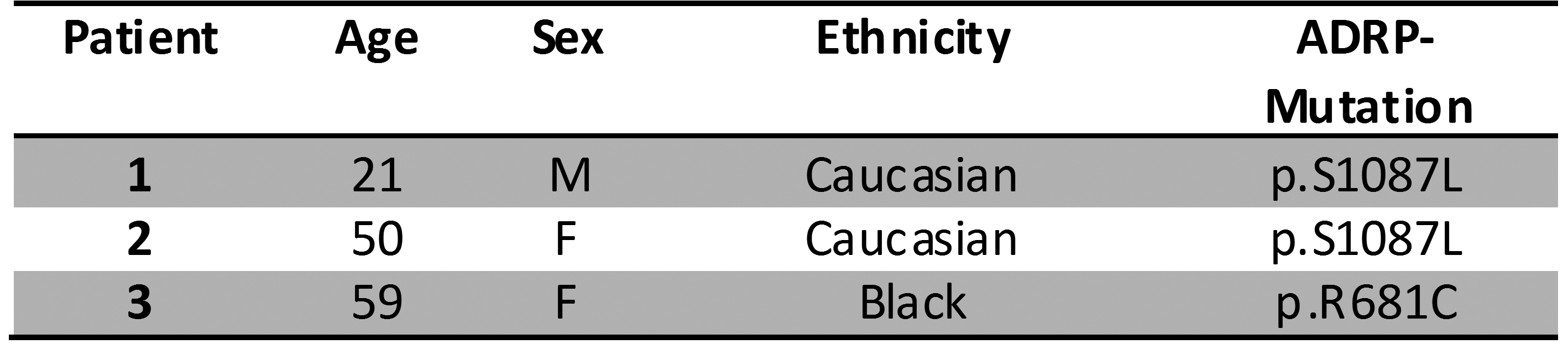

Supplement: S1 Table — Mean ages of patients and healthy donors were matched (43.3 vs. 43.0). (TIF) [file ppat.1005772.s017.tif]
