# Supplementary figures and images for: Correction: Spliceosome SNRNP200 Promotes Viral RNA Sensing and IRF3 Activation of Antiviral Response
Source: PLoS Pathog. 2017 Jan 24;13(1):e1006174. doi: 10.1371/journal.ppat.1006174 (PMC5261561; doi:10.1371/journal.ppat.1006174)

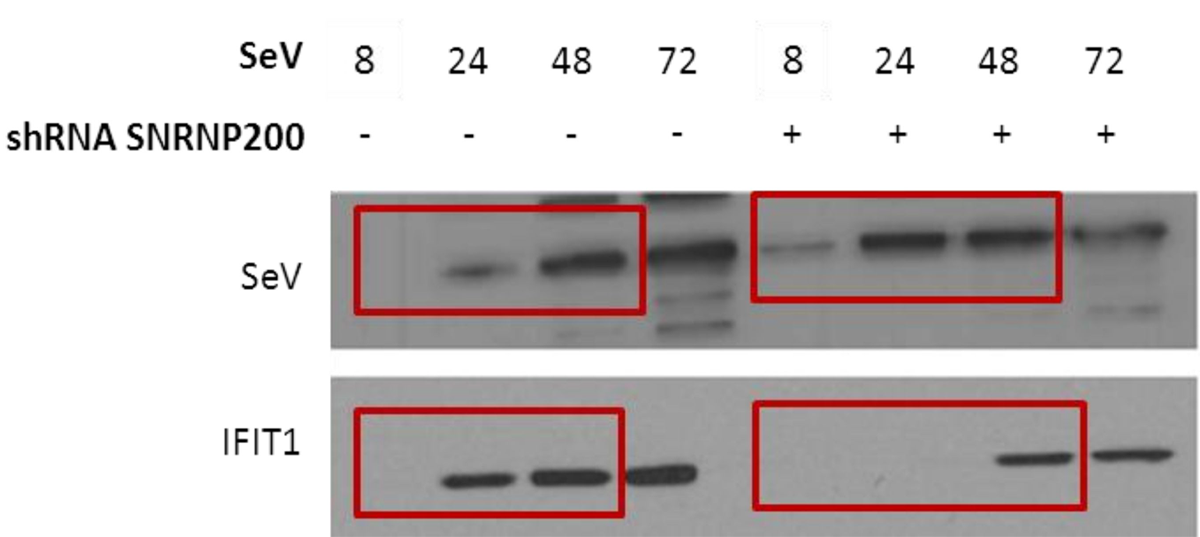

Supplement: S1 Fig — Immunoblot analysis of HEK 293T cells infected with SeV for 8, 24, 48 or 72 hours following treatment with shNT or shSNRNP200 for three days. SeV and IFIT1 proteins are resolved by immunobloting at the indicated time. For clarity, the lane for the 72-hour time point infection was removed of Fig 1C. Red boxes indicate the cropped area used in Fig 1C. The immunobloting of actin is not available as the raw data could not be retrieved. (TIF) [file ppat.1006174.s001.tif]

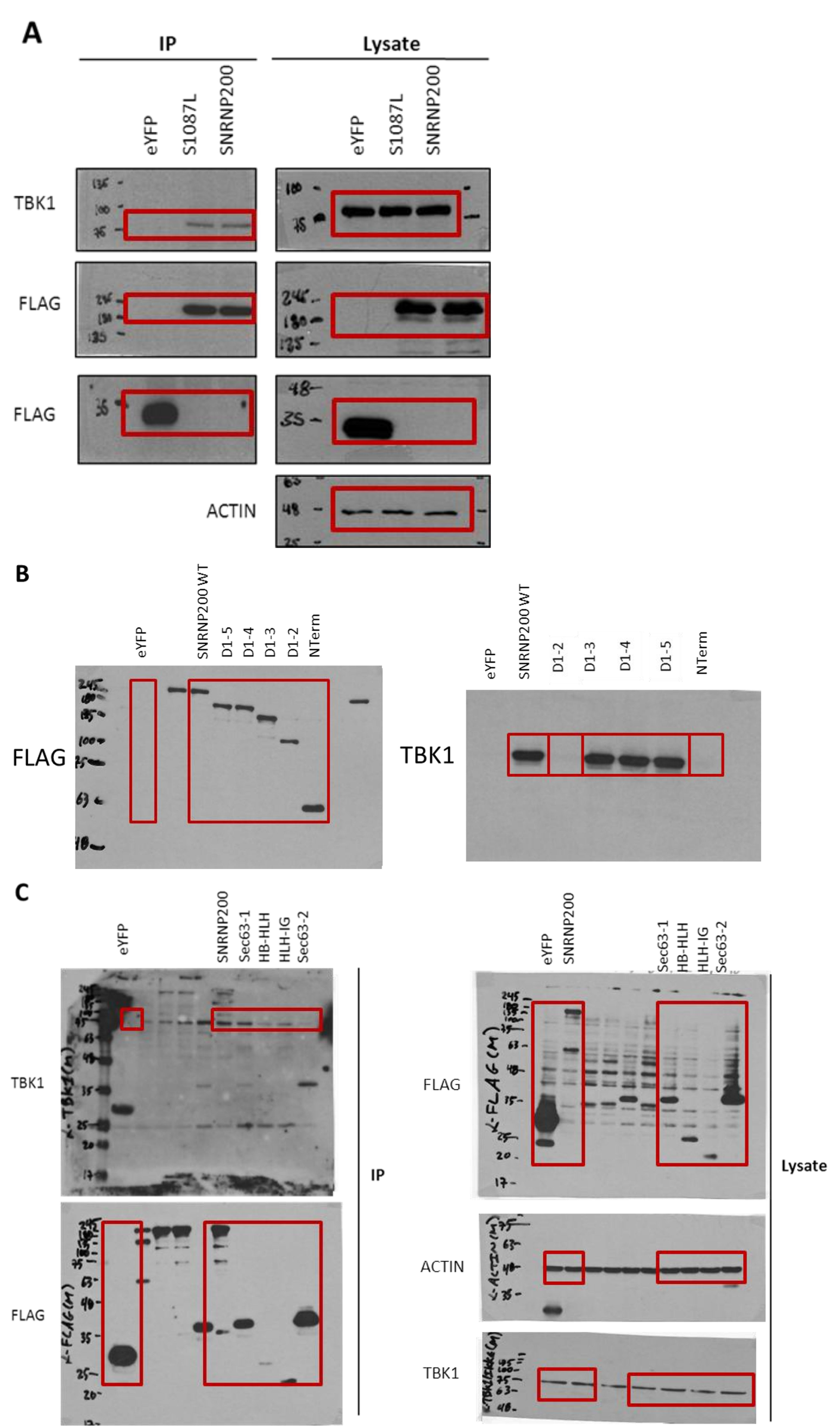

Supplement: S2 Fig — (A) HEK 293T cells are transfected with FLAG-eYFP (control), FLAG-SNRNP200 or FLAG-SNRNP200 S1087L mutant expressing plasmids for 48 hours. Cell lysates are prepared following 16 hours of SeV infection and subjected to immunoprecipitation with anti-FLAG antibodies. Cell lysates and immune complexes are resolved by immunobloting analysis using anti-FLAG and anti-TBK1 antibodies. Red boxes indicate the cropped area used in Fig 6A. (B) Immunoprecipitation of FLAG-SNRNP200 C-terminal deletion mutants. HEK 293T cells are transfected with FLAG-eYFP (control), FLAG-SNRNP200 and FLAG-deletion mutants expressing plasmids for 48 hours. Cell lysates are prepared following 16 hours of SeV infection and subjected to immunoprecipitation with anti-FLAG antibodies. Immune complexes are resolved by immunobloting analysis using anti-FLAG and anti-TBK1 antibodies. Left. Cropping was done on the blots to remove lanes of the larger experiment. Right. Cropping was done to enhance clarity and match the presentation of the overall figure. Red boxes indicate the cropped area used in Fig 6B. (C) Immunoprecipitation of FLAG-SNRNP200 Sec63-1, HB-HLH or HLH-IG subdomains of Sec63-1 and Sec63-2 are performed and analyzed as indicated in (A). Cropping was done on the blots from a larger experiment to remove unnecessary lanes and enhance clarity. Red boxes indicate the cropped area used in Fig 6C. (TIF) [file ppat.1006174.s002.tif]

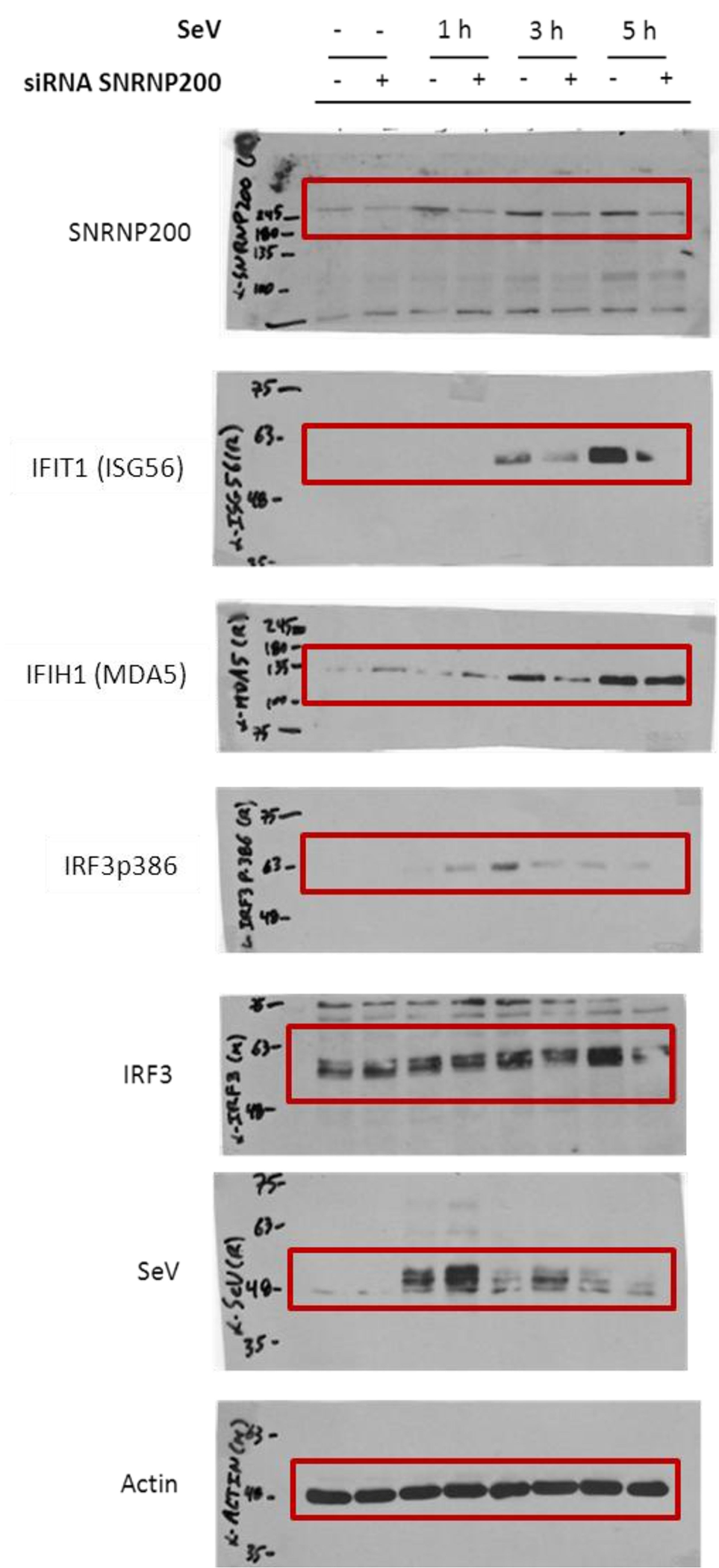

Supplement: S3 Fig — (A-B) MDM are transfected with a pool of siRNA targeting SNRNP200 for 48 hours and infected with SeV. At 1, 3 and 5 hours post-infection, cells are harvested and selected proteins (SNRNP200, IFIT1, IFIH1, IRF3, IRF3-p386, SeV and actin) are resolved by immunobloting of cells lysates and compared to control cells treated with scrambled siRNA. Red boxes indicate the cropped area used in Fig 8A-B. (TIF) [file ppat.1006174.s003.tif]
